# Efficacy of Traditional Chinese Medicine Tonifying Kidney (Bushen) and Activating Blood (Huoxue) Prescription for Premature Ovarian Insufficiency: A Systematic Review and Meta-Analysis

**DOI:** 10.1155/2020/1789304

**Published:** 2020-04-21

**Authors:** Hui-Fang Li, Qi-Hong Shen, Wen-Jun Chen, Wei-Min Chen, Zhang-Feng Feng, Li-Ying Yu

**Affiliations:** ^1^Department of TCM Gynecology, Tongxiang Maternal and Child Health-Care Center, Tongxiang, Zhejiang, China; ^2^Department of Anesthesiology, Affiliated Hospital of Jiaxing University, The First Hospital of Jiaxing, Jiaxing, Zhejiang, China; ^3^Department of Gynecology, Zhejiang Province Hospital of TCM, Hangzhou, Zhejiang, China; ^4^Department of Gynecology, Tongxiang Maternal and Child Health-Care Center, Tongxiang, Zhejiang, China

## Abstract

**Objective:**

We carried out this systematic review and meta-analysis to evaluate the effectiveness of TKABP on POI.

**Methods:**

The following eight databases were searched from the establishment to September 30, 2019, for randomized controlled trials (RCTs): PubMed, Embase, Cochrane Library, Web of Science, China National Knowledge Infrastructure (CNKI), the Chinese BioMedical database (CBM), Chinese Scientific Journal Database (VIP), and the Wanfang database. The quality of evidence was estimated by the Grading of Recommendations Assessment, Development, and Evaluation (GRADE).

**Results:**

Twenty-three RCTs involving 1712 patients with POI were included. Compared to hormone therapy (HT) groups, TKABP groups showed a significantly higher total effective rate (RR: 1.10; 95% CI: 1.04–1.17; *P* < 0.01, *I*^2^ = 32%). In addition, TKABP groups revealed a better improvement in terms of serum follicle-stimulating hormone (FSH) levels, serum estradiol (E_2_) levels, peak systolic velocity (PSV) of ovarian stromal blood, and Kupperman index (KI) score. However, serum luteinizing hormone (LH) levels and ovarian volume (OV) showed no significant statistical difference. Subgroup analyses showed that herbal paste and 3 months of treatment duration had a greater effect on the improvement of hormone levels. Besides, the occurrence of related adverse events in TKABP groups was lower than that in HT groups.

**Conclusions:**

Our review suggests that TKABP appears to be an effective and safe measure for patients with POI, and the herbal paste may be superior. However, the methodological quality of included RCTs was unsatisfactory, and it is necessary to verify its effectiveness with furthermore standardized researches of rigorous design.

## 1. Introduction

Premature ovarian insufficiency (POI) is currently considered the most apposite term to denote the loss of ovarian function caused by an abnormal and accelerated depletion of ovarian reserve in women before the age of forty [1]. It was characterized with the declining levels of normal hormonal and reproductive function [2]with the prevalence in the general population being approximately 1% [1]. POI is a frustrating gynecological endocrine disease triggered by highly heterogeneous causes, including socioeconomic status [3], autoimmune aspect [4], and prenatal ethanol exposure [5]. Previous studies had reported that women with POI had more medical issues than natural menopausal women, such as overall mortality [6], lipid disorders, cardiovascular diseases [7], osteoporosis [8], psychiatric diseases, and other adverse health complications [9,10], which could have potentially devastating effect upon woman's health, physically and psychologically. Despite the fact that an increasing number of women worldwide are suffering from POI, the exact conclusions about the therapy of POI are still rare. Hormone therapy (HT), one of the most commonly methods used to treat POI, only aims to relieve the signs and symptoms of POI and may cause hepatic damage, vascular conditions, and cancer risk with long-term treatment [11].

Based on the traditional Chinese medicine theory, kidney deficiency and blood stasis are important pathogenesis of POI. Tonifying kidney (bushen) and activating blood (huoxue) is a traditional Chinese medicine treatment, which was widely used in the treatment of congestion-related diseases [12–14]. Previous researches reported that tonifying kidney (bushen) and activating blood (huoxue) treatment acts a pivotal part in the management of POI [15, 16]. Zeng et al. [17] found that bushen huoxue recipe was superior to HT for treating POI. A recent meta-analysis indicated bushen huoxue Chinese medicine can reduce the symptoms of patients suffering from POI [18]. However, the included studies were not complete, and the sample size was relatively small. Thus, we conducted this systematic review and meta-analysis for random controlled trials to evaluate the efficacy and safety of TKABP for the treatment of POI.

## 2. Materials and Methods

We reported this systematic review and meta-analysis following the Preferred Reporting Items for Systematic Reviews and Meta-Analyses (PRISMA) guidelines [19]. The number of registration in PROSPERO is CRD42019148035.

### 2.1. Search Strategy

Computer retrieved clinical studies databases including PubMed, Embase, Cochrane Library, Web of Science, China National Knowledge Infrastructure (CNKI), the Chinese BioMedical database (CBM), Chinese Scientific Journals Database (VIP), and the Wanfang database with no limitations on language and publication status. Each database was searched from their establishment to September 30, 2019. We made the retrieval formula according to the PICOS strategy. For Chinese databases, we took CNKI as an example, and the specific retrieval formula was SU = (Chinese medicine + traditional Chinese medicine + Chinese herb + bushen + huoxue) AND (premature ovarian failure + primary ovarian insufficiency + premature ovarian insufficiency + POI + POF) AND (randomization + randomized controlled + random grouping + RCT + clinical research). For other databases, we took PubMed as an example, and the search strategy was reported in Supplement Digital. These search terms will be precisely translated for other databases. We also manually searched the references of the original and reviewed articles for possible related studies to supplement the relevant literature.

### 2.2. Selection Criteria

#### 2.2.1. Inclusion Criteria

The inclusion criteria include the following: (1) population: patients diagnosed with POI, regardless of ethnicity or nationality; (2) intervention: the therapy of Chinese herbal medicine tonifying kidney and activating blood was clearly stated in the trial group with no limitation in prescription name, dosage form, dosage, and course of treatment; (3) comparison: the comparison that tonifying kidney (bushen) and activating blood (huoxue) prescription only versus HT, no treatment, placebo, or sham treatment was investigated; (4) outcome: reporting the effect of TKABP for POI; and (5) study design: random controlled trial.

#### 2.2.2. Exclusion Criteria

The exclusion criteria include the following: (1) animal experiments; (2) duplicated articles; (3) unable to get original data; (4) the composition of prescription is not clear; (5) other traditional Chinese medicine treatments such as acupuncture, edema, and massage.

### 2.3. Outcome Indicators

#### 2.3.1. Primary Outcome Measures

Primary outcomes are the total effective rate, serum estradiol (E_2_), serum follicle-stimulating hormone (FSH), and serum luteinizing hormone (LH) levels. For studies that classified treatment effect into different grades while the total effective rate was not reported, we combined the effective grades into “total effective” for analysing.

#### 2.3.2. Secondary Outcome Measures

The second outcomes are peak systolic velocity (PSV) of ovarian stromal blood, Kupperman index (KI) score, ovarian volume (OV), and incidence of adverse events.

### 2.4. Data Extraction

Two authors (Hui-fang Li and Wen-jun Chen) independently extracted the following information by a predesigned and standardized data extraction form: first author, year of publication, sample size, age, course of disease, treatment interventions and control groups, treatment duration, and outcomes. Any conflict was resolved by a third author (Qi-hong Shen).

### 2.5. Quality Assessment

The risk of bias for the included trial was independently evaluated by two researchers (Wei-min Chen and Zhang-feng Feng) in reference to the Cochrane Handbook. We evaluated the following criteria: random sequence generation, allocation concealment, blinding of participants and personnel, blinding of outcome assessments, incomplete outcome data, selective reporting, and other biases. Each study was classified into low, high, or unclear. If there was a disagreement, we referred to the views of the third researcher (Qi-hong Shen).

### 2.6. GRADE Evaluation

The quality of outcome was evaluated by GRADE (Grading of Recommendations Assessment, Development, and Evaluation) according to the following criteria: study design, risk of bias, rating inconsistency in results, rating indirectness of evidence, and others. The quality of evidence was classified as high, moderate, low, or very low.

### 2.7. Statistical Analysis

We conducted this meta-analysis by using Review Manager 5.3 statistical software. Regarding the study outcomes, relative risk (RR) with 95% confidence interval (CI) was used for binary variables, while weighted mean difference (WMD) and 95% CI were presented for continuous variables. Cochrane's *P* values and *I*^2^ were tested to examine heterogeneity among the studies. High heterogeneity most likely existed due to the clinical and methodological factors, so the random effect model was adopted in this meta-analysis even *I*^2^ was small. Subgroup analysis was performed based on duration treatment (3 months vs more than 3 months) and dosage form (herbal paste vs herbal decoction) for primary outcomes. Funnel plots were tested for assessing the publication bias when the number of trials ≥ 10. In addition, sensitivity analysis was performed by sequentially deleting trials to check the stability of the primary outcomes.

## 3. Result

### 3.1. Search Results

**Figure 1:**
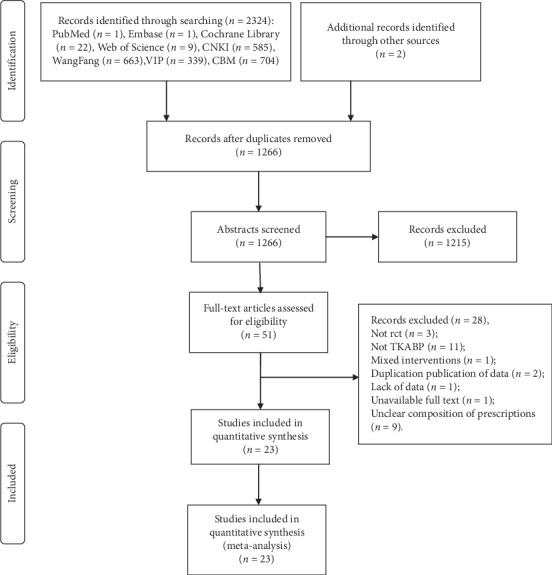
The search process of included studies.

Initially, 2326 relevant studies were identified. After excluding duplicate studies, we scanned 1266 studies based on their abstracts and titles. Then, 51 articles were evaluated by full text. We also excluded 28 trials for the following reasons: eleven non-TKABP studies, nine articles with unclear composition of prescription, three studies were not RCT, two articles with duplicate publication of data, one article with mixed interventions of acupuncture, one article was lack of duration treatment, and another one article with unavailable full text. Eventually, 23 studies were included in our system review [15–17, 20–39]. The search process was displayed in Figure 1.

### 3.2. Study Characteristics

Table 1 shows the details of the included studies. Of these trials, all of them were published in China. A total of 1712 patients with POI were contained in these studies, including 881 in the TKABP group and 831 in the control group. The diagnosis of POI was clearly identified in 17 studies [15–17, 21–23, 25–27, 30, 32–36, 38, 39] and not mentioned in 6 studies [20, 24, 28, 29, 31, 37]. Nineteen studies were treated with pure herbal decoction [15–17, 20–27, 29–31, 34–36, 38, 39], one study was applied herbal decoction plus Chinese patent medicine [28], two studies were cured with herbal paste [32, 37], and one study included both herbal decoction and herbal paste groups [33]. Patients in the control group were all treated with HT. The treatment duration was set for 3 months in 7 studies [16, 17, 21–23, 28, 29, 32–35, 37–39], 6 months in 7 studies [15, 24–27, 30, 31],and 9 months in 2 studies [20, 36]. Of these 23 studies, 20 trials presented the total effective rates [15, 16, 20–29, 31–36, 38, 39]; 19 trials reported FSH, E_2_, and LH levels [15–17, 21, 22, 24, 26–28, 30–39], 2 trials reported PSV [26, 30], 7 trials mentioned KI [16, 21, 26, 31, 34, 35, 38], 3 trials stated OV [15, 30, 35], and 14 trials mentioned adverse events [15–17, 20–22, 25, 29, 31–35, 39]. The composition of prescription in the included studies is shown in Supplement Table 1.

### 3.3. Risk of Bias Assessment

**Figure 2:**
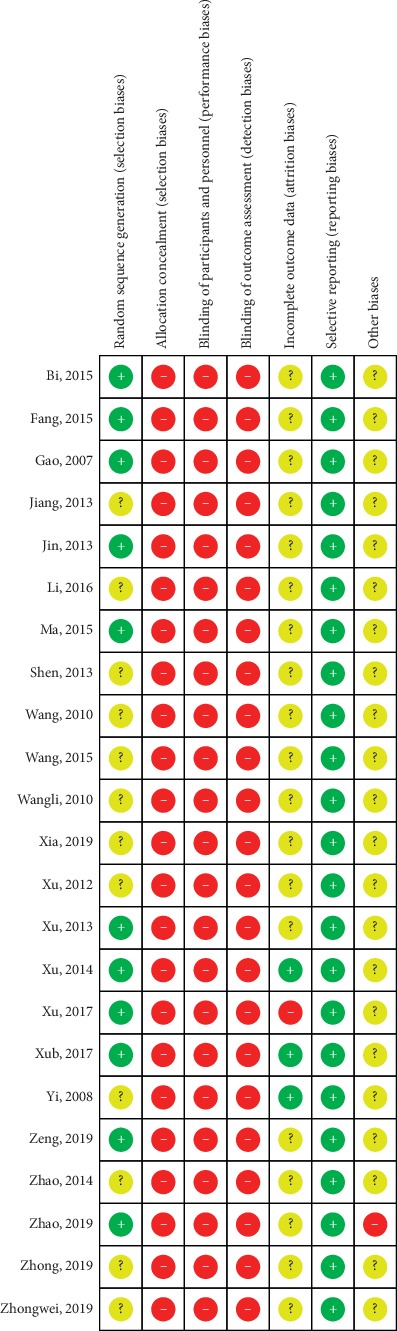
The risk of bias for included studies.

Although 23 studies mentioned randomized, just 11 clearly reported the random method (random number table) [15–17, 20–22, 25, 31–33, 37]. None of the trials reported any concealed allocation or blinding of patients and investigators. Three trials indicated the number and reasons of dropouts [31, 33, 35]; no selective reporting was reported. The risk of bias summary is shown in Figure 2.

## 4. Outcome Measures

### 4.1. Primary Outcomes

**Figure 3:**
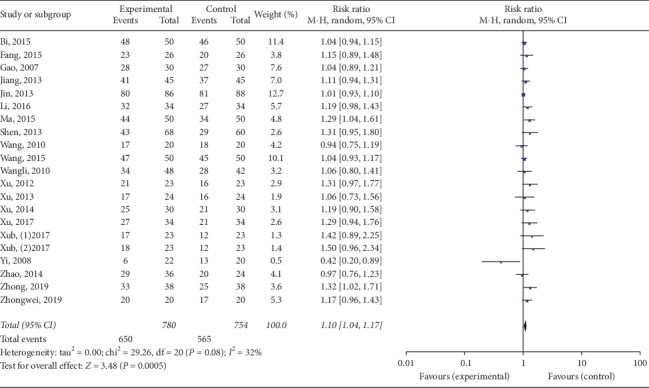
Forest plot for total effective rate between TKABP and control group.

**Figure 4:**
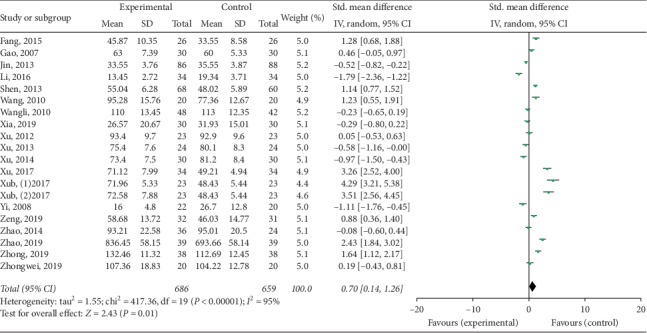
Forest plot for E_2_ level between TKABP and control group.

**Figure 5:**
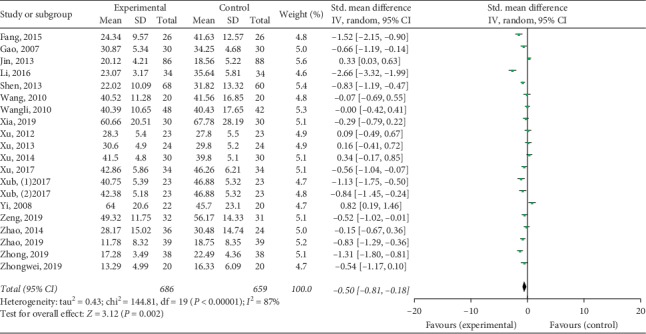
Forest plot for FSH level between TKABP and control group.

**Figure 6:**
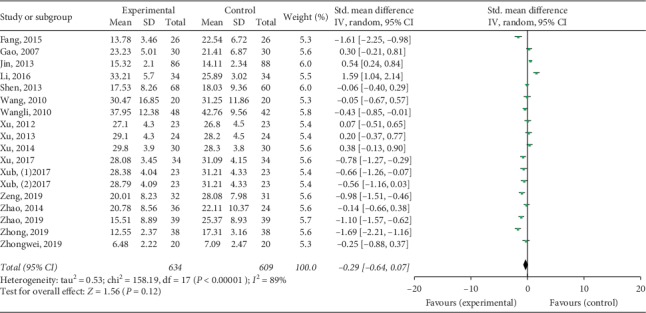
Forest plot for LH level between TKABP and control group.

Twenty studies mentioned the treatment effect. TKABP led to a significantly higher total effective rate (RR: 1.10; 95% CI: 1.04, 1.17; *P* < 0.001, *I*^2^ = 32%, Figure 3). Serum E_2_ and FSH levels were assessed in 19 trials; LH levels were measured in 17 trials. The pooled data of meta-analysis demonstrated that the E_2_ levels were significantly higher (SMD: 0.70; 95% CI: 0.14, 1.26; *P* < 0.05, *I*^2^ = 95%, Figure 4), while FSH levels (SMD: −0.50; 95% CI: −0.81, −0.18; *P* < 0.05, *I*^2^ = 95%, Figure 5) were significantly lower in the TKABP group. The result showed no significant difference about LH levels (SMD: −0.29; 95% CI: −0.64, 0.07; *P*=0.12, *I*^2^ = 89%, Figure 6).

### 4.2. Secondary Outcomes

**Figure 7:**
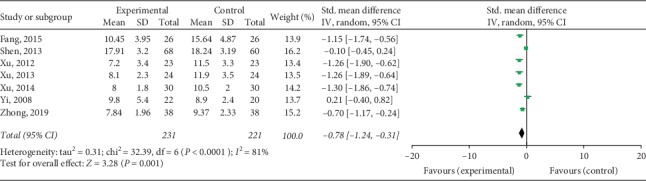
Forest plot for Kupperman scores between TKABP and control group.

**Figure 8:**
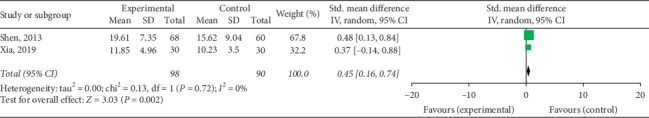
Forest plot for PSV of ovarian stromal blood between TKABP and control group.

**Figure 9:**
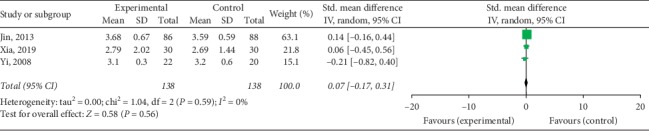
Forest plot for OV between TKABP and control group.

Compared with controls, patients treated with TKABP had significantly lower Kupperman scores (SMD: −0.78; 95% CI: −1.24, −0.31; *P* < 0.05, *I*^2^ = 81%, Figure 7) and significantly higher PSV of ovarian stromal blood (SMD: 0.45; 95% CI: 0.16, 0.74; *P* < 0.05, *I*^2^ = 0%, Figure 8). No significant difference about OV was spotted between the trial and control groups (SMD: 0.07; 95% CI: −0.17, 0.31; *P*=0.56, *I*^2^ = 0%, Figure 9).

**Figure 10:**
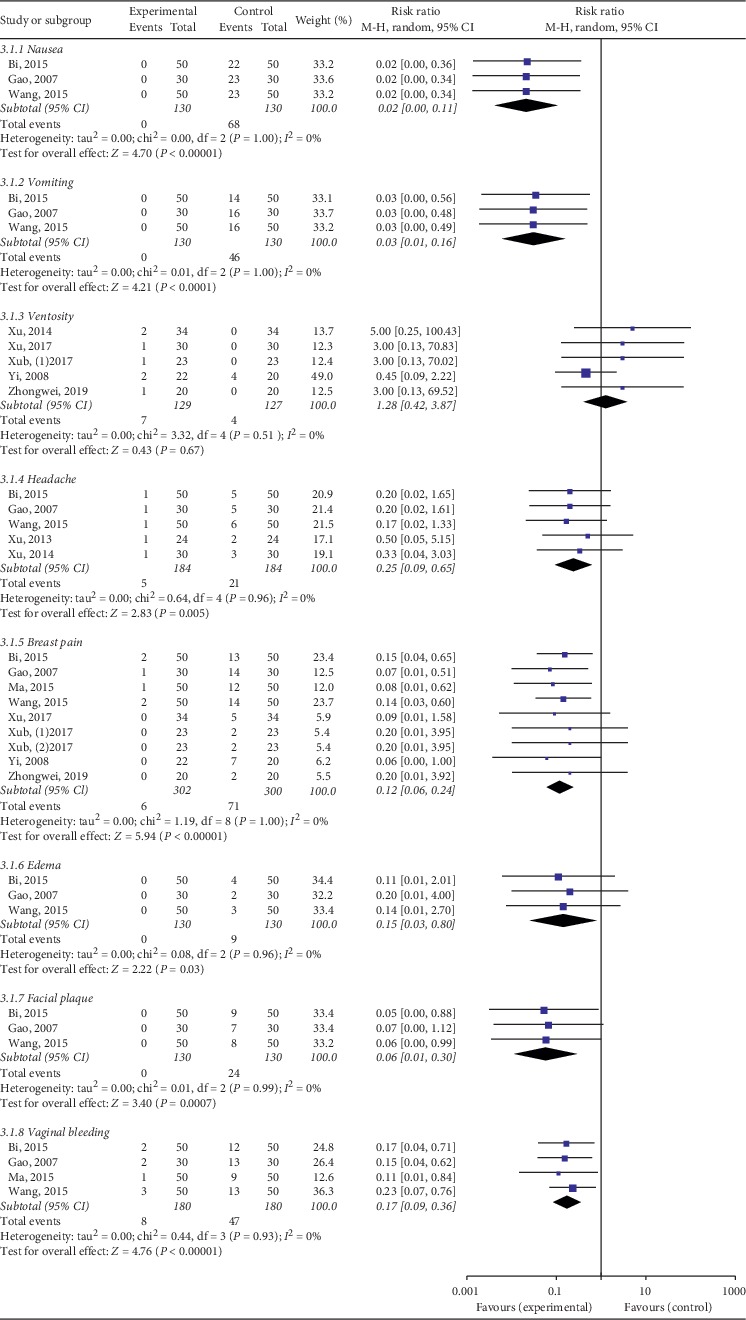
Forest plot for side effects between TKABP and control group.

No significant difference about occurrence of ventosity was revealed (RR 1.28, 95% CI 0.43–3.87, *P*=0.67, *I*^2^ = 0%, Figure 10). Other adverse effects, including nausea, vomiting, headache, breast pain, edema, facial plaque, and vaginal bleeding, had no significantly difference reported (Figure 10).

### 4.3. Subgroup Analysis

We performed subgroup analyses to further analyze the source of significant heterogeneity. Subgroup analyses showed that the total effective rate had no significant difference between patients who received herbal paste or herbal decoction (Supplement Figure 1). However, total effective rate in patients with 3 months of treatment duration was significantly higher (Supplement Figure 2). In addition, forest plot demonstrated that herbal paste and 3 months of treatment duration led to a significant better improvement in terms of serum E_2_, FSH, and LH levels, and the heterogeneity significantly reduced (Supplement Figures 3–8). The results of subgroup analyses are summarized in Table 2.

### 4.4. Publication Bias and Sensitivity Analysis

Funnel plots for total effective rate and serum E_2_, FSH, and LH levels were in symmetric distribution, which indicated publication bias was not existed (Supplement Figures 9–12). Sensitivity analysis was performed for the total effective rate, and the effect estimate remained unchanged, which indicated the robustness of the pooled results (Supplement Figures 1–3).

### 4.5. GRADE Evaluation

The quality of evidence was low for total effective rate, serum E_2_, FSH, and LH levels, and Kupperman score. The GRADE level of evidence was moderate for OV, PSV of ovarian stromal blood, and complications. Summary of GRADE evaluation is shown in Table 3.

## 5. Discussion

Our study demonstrated that TKABP increases the total effective rate of POI, improves the serum E_2_ and FSH levels, PSV of ovarian stromal blood, and Kupperman index, and decreases the incidence of adverse effects. The quality of evidence was moderate and low. In addition to the above effects, herbal paste of TKABP and 3 months treatment might be more effective.

According to TCM theory, the etiology and pathogenesis of POI are always dominated by the deficiency of kidneys, which store essence and dominate reproduction, including “qi deficiency,” “yin deficiency,” and “yang deficiency.” Qi deficiency patients are unable to promote blood operation, yin deficiency patients with pulse path rigidity, and yang deficiency with pulse stagnation, which may lead to blood stasis. Kidney deficiency and blood stasis also affect and transform each other. Therefore, the focus of treatment is to regulate hormone levels and improve ovarian function by tonifying kidney and promoting blood circulation. Many studies whether they were clinical or animal researches have shown that Chinese nourishing kidney and activating blood herbs, such as Prepared radix rehmanniae, dodder, Chinese yam, safflower, Salvia, and Lycium barbarum, have the effect of phytoestrogen [40, 41] and also can regulate the reproductive axis in dual directions, enhance or regulate the immune function, and prevent osteoporosis [42–44]. In this meta-analysis, we found that TKABP could significantly accelerate the peak systolic velocity of ovarian stromal blood, which can alleviate blood stasis to improve the blood supply of ovaries.

Subgroup analysis for total effective rate and hormone levels showed that the TKABP group was better than the HT group in 3 months course studies. However, in more than 3 months course studies, there was no statistically significant difference between the two groups. It may be related to the poor long-term adherence to Chinese medicine for patients [45]. The study also showed that compliance of researchers in clinical trials of TCM may be affected with the extension of treatment time [46]. Another subgroup analysis based on dosage form showed that the total effective rate had no significant difference. However, herbal paste had a significantly better improvement of serum E_2_, FSH, and LH levels, and the heterogeneity significantly reduced, which indicated that herbal paste might be better than the herbal decoction for treating POI. The results of previous clinical studies also showed that the herbal paste had obvious advantages over the traditional decoction, such as stable property, easy preservation, convenient administration, and long-lasting effect, which led to a better compliance among patients [47]. However, the number of studies that reported herbal paste was really small, and we should be more careful in promoting this result.

A recent meta-analysis focused on the effect of TKABP for patients with premature ovarian failure. There are some different aspects to our study: first, the previous meta-analysis only included 12 RCTs, while 23 trials were analyzed in our study; second, some trials in the previous meta-analysis did not clearly report the ingredients of TKABP, which may cause a little bias; third, we assessed the quality for the evidence by GRADE. So, it is necessary for us to conduct this meta-analysis.

## 6. Limitations

In addition, some limitations in this study should be acknowledged. First, the included studies had low quality due to an unclear allocation concealment, selective bias, attrition bias, and blinding methods, and all studies do not preestimate the sample size. Second, the ingredient of TKABP was different among studies, which might result in bias. Third, although we searched the studies without language restriction, all the publication regions were in China. Fourth, studies with negative results may have been published with a lower frequency, leading to publication bias. Fifth, the criteria for the efficacy of each study was inconsistent. As a result, the evaluation had certain subjectivity and difference, which affected the accuracy and stability of the outcome.

## 7. Conclusion

In summary, our results show that TCM therapy tonifying kidney and activating blood may be a safe and effective treatment for POI and could be considered as an alternative treatment to conventional therapy. In addition, the herbal paste may be a better choice. However, due to the relatively low quality of the included studies, we should be in more caution to promote this result. We should standardize and unify the diagnosis and treatment standards, and a well-designed, multicenter, and large-sample study was needed to ensure the scientific, objective, and reliable conclusions of the research in the future clinical research so as to make the results more convincing and provide clinical evidence for the treatment of POI with TCM tonifying kidney and activating blood prescription.

## Figures and Tables

**Table 1:** The detail of included studies.

Studies	Sample size (*n*)		Age (*y*)		Course of disease		Intervention measures		Duration treatment (mos)	Main outcomes
	Trial group	Control group	Trial group	Control group	Trial group	Control group	Trial group	Control group		
Jin, 2013	86	88	33.25 ± 3.11	34.11 ± 3.31	147.99 ± 21.85 days	148.11 ± 21.89 days	Bushenhuoxue decoction	A + B	6	①②③④⑥⑧
Xu, 2013	24	24	31.8 ± 3.6	31.6 ± 3.7	1.5 ± 0.4 yrs	1.6 ± 0.5 yrs	Bushentiaojing decoction	A + B	3	①②③④⑦⑧
Bi, 2015	50	50	34.5 ± 3.6	33.9 ± 3.2	17.8 ± 9.4 mos	18.1 ± 9.6 mos	Bushentiaochong decoction	A + B	9	①⑧
Zeng, 2019	32	31	28.63 ± 6.49	31.06 ± 6.61	2.21 ± 1.07 yrs	2.02 ± 0.92 yrs	Huoxuezishen decoction	E	3	②③④⑧
Fang, 2015	26	26	32.6 ± 2.9	33.2 ± 3.9	12.5 ± 3.7 mos	13.3 ± 4.2 mos	Bushenhuoxue decoction	C + D	3	①②③④⑦⑧
Gao, 2007	30	30	33.033 ± 3.017	32.96 ± 4.563	18.27 ± 9.96 mos	19.23 ± 9.71 mos	Bushentiaochong decoction	A + B	3	①②③④⑧
Jiang, 2013	45	45	35.4	34.9	2.8 yrs	2.6 yrs	Yishenguijing decoction	A + B	3	①
Li, 2016	34	34	Not mentioned	Not mentioned	Not mentioned	Not mentioned	Bushenhuoxue decoction	B + C	6	①②③④
Ma, 2015	50	50	28.70 ± 9.53	28.21 ± 6.09	13.86 ± 7.51 mos	14.71 ± 7.20 mos	Bushen decoction	C	6	①⑧
Shen, 2013	68	60	32.45 ± 4.16	31.68 ± 3.97	Not mentioned	Not mentioned	Zishenhuoxue decoction	E	6	①②③④⑤⑦
Wang, 2010	20	20	34.53 ± 4.32	33.72 ± 6.48	23.25 ± 13.42 mos	23.25 ± 13.42 mos	Bushenhuoxue decoction	B + C	6	①②③④
Wang, 2010	48	42	Not mentioned	Not mentioned	Not mentioned	Not mentioned	Bushentiaojing decoction + Dahuang Zhechong pill	B + C	3	①②③④
Wang, 2015	50	50	33.2 ± 3.4	32.6 ± 3.5	18.2 ± 9.1 mos	19.2 ± 9.4 mos	Bushenchongtiao decoction	B + C	3	①⑧
Xia, 2019	30	30	37.13 ± 4.45	35.4 ± 4.89	3.03 ± 1.58 yrs	3.33 ± 1.74 yrs	Bushenhuoxue decoction	B + C	6	②③⑤⑥
Xu, 2014	30	30	36.5 ± 2.8	36.0 ± 2.7	1.5 ± 0.4 yrs	1.6 ± 0.5 yrs	Bushentiaojing decoction	A + B	6	①②③④⑦⑧
Xu, 2017	34	34	38.03 ± 0.83	37.53 ± 0.96	1.91 ± 0.75 yrs	1.94 ± 0.74 yrs	Bushentiaojing paste	A + B	3	①②③④⑧
Xu, 2017	23	23	37.52 ± 1.16	37.78 ± 1.09	1.91 ± 0.73 yrs	1.96 ± 0.71 yrs	Bushentiaojing decoction	A + B	3	①②③④⑧
Xu, 2017	23	23	38.04 ± 1.11	37.78 ± 1.09	2.08 ± 0.85 yrs	1.96 ± 0.71 yrs	Bushentiaojing paste	A + B	3	①②③④⑧
Xu, 2012	23	23	29.1 ± 3.4	28.7 ± 3.1	1.6 ± 0.3 yrs	1.7 ± 0.4 yrs	Bushentiaojing decoction	F	3	①②③④⑦⑧
Yi, 2008	22	20	34.9 ± 3.5	33.8 ± 3.9	18.7 ± 6.9 mos	19.1 ± 7.9 mos	Tiaojingkangshuai			
							Decoction	A + B	3	①②③⑥⑦⑧
Zhao, 2014	36	24	33.04 ± 4.3	32.89 ± 4.03	1.90 ± 1.52 yrs	2.18 ± 1.36 yrs	Fuchao decoction	B + C	9	①②③④
Zhao, 2019	39	39	23.55 ± 5.55	23.64 ± 5.44	1.61 ± 0.61 yrs	1.58 ± 0.22 yrs	Bushentiaojing paste	C + D	3	②③④
Zhong, 2019	38	38	34.12	34.10	9.29 ± 4.35 mos	9.30 ± 4.40 mos	Zishenhuoxue decoction	A + B	3	①②③④⑦
Zhong Wei, 2019	20	20	36.0 ± 2.02	36.2 ± 1.94	6.15 ± 1.93 mos	6.10 ± 1.62 mos	Bushenhuoxue decoction	C + D	3	①②③④⑧

Abbreviation: mos, months; yrs, years; A, conjugated estrogen; B, medroxyprogesterone acetate (MPA); C, estradiol valerate; D, progesterone capsule; E, Climen (combination of estradiol valerate and cyproterone acetate); F, Marvelon (combination of desogestrel and ethinylestradiol). ① The total effective rate; ② serum estradiol (E_2_) levels; ③ serum follicle-stimulating hormone (FSH) levels; ④ serum luteinizing hormone (LH) levels; ⑤ peak systolic velocity (PSV) of ovarian stromal blood; ⑥ ovarian volume (OV); ⑦ Kupperman index (KI) score; and ⑧ adverse events.

**Table 2:** Subgroup analysis for primary outcomes.

	Studies	MD/SMD/RR	95 CI	*P*	*I* ^*2*^ (%)
*Total effective rate*					
3 M	14	1.14	(1.06, 1.22)	<0.05	21
<3 M	6	1.04	(0.97, 1.02)	0.24	21
*E*_*2*_					
3 M	12	1.19	(0.44, 1.94)	<0.05	95
<3 M	7	−0.18	(−0.94, 0.57)	0.63	94
*FSH*					
3 M	12	−0.53	(−0.86, −0.91)	<0.05	80
<3 M	7	−0.46	(1.10, 0.19)	0.16	93
*LH*					
3 M	11	−0.62	(−0.99, −0.26)	<0.05	81
<3 M	6	0.37	(−0.09, 0.83)	0.11	84
*Total effective rate*					
Herbal decoction	19	1.09	(1.03, 1.15)	<0.05	27
Herbal paste	2	1.10	(1.04, 1.17)	<0.05	0
*E*_*2*_					
Herbal decoction	17	0.29	(−0.21, 0.79)	0.26	94
Herbal paste	3	3.00	(2.32, 3.67)	<0.05	59
*FSH*					
Herbal decoction	17	−0.46	(−0.82, −0.09)	<0.05	88
Herbal paste	3	−0.73	(−1.03, −0.44)	<0.05	0
*LH*					
Herbal decoction	15	−0.19	(−0.59, 0.21)	0.36	90
Herbal paste	3	−0.85	(−1.14, −0.55)	<0.05	0

**Table 3:** Summary of meta-analysis results and grade evaluation.

Index	Number of included studies	SMD/MD/RR (95% CI)	*P* value	*I* ^2^ value (%)	GRADE
Total effective rate	20	1.10 (1.04, 1.17)	<0.05	32	⊕⊕○○Low
*E*_2_	19	0.70 (0.14, 1.26)	<0.05	95	⊕⊕○○Low
FSH	19	−0.50 (−0.81, −0.18)	<0.05	87	⊕⊕○○Low
LH	17	−0.29 (−0.64, 0,09)	0.12	89	⊕⊕○○Low
Ovarian volume	3	0.07 (−0.17, 0.31)	0.56	0	⊕⊕⊕○Moderate
Kupperman score	7	−0.78 (−1.24, -0.31)	<0.05	81	⊕⊕○○Moderate
PSV of ovarian stromal blood	2	0.45 (0.16, 0.74)	<0.05	0	⊕⊕⊕○Moderate
Nausea	3	0.02 (0.00, 0.11)	<0.05	0	⊕⊕⊕○Moderate
Vomiting	3	0.03 (0.01, 0.16)	<0.05	0	⊕⊕⊕○Moderate
Ventosity	5	1.28 (0.42, 3.87)	0.67	0	⊕⊕⊕○Moderate
Headache	5	0.25 (0.09, 0.65)	<0.05	0	⊕⊕⊕○Moderate
Breast pain	8	0.12 (0.06, 0.24)	<0.05	0	⊕⊕⊕○Moderate
Edema	3	0.15 (0.03, 0.80)	<0.05	0	⊕⊕⊕○Moderate
Vaginal bleeding	4	0.17 (0.09, 0.36)	<0.05	0	⊕⊕⊕○Moderate

## Supplementary Materials

Supplementary Materials Supplement Digital: PubMed retrieval strategy. Supplement Table 1: composition of prescription in the included studies. Supplement Table 2: PRISMA checklist. Supplement Figure 1: subgroup analysis for total effective rate with different dosage forms. Supplement Figure 2: subgroup analysis for total effective rate with different duration treatment. Supplement Figure 3: subgroup analysis for E_2_ with different dosage forms. Supplement Figure 4: subgroup analysis for FSH with different dosage forms. Supplement Figure 5: subgroup analysis for LH with different dosage forms. Supplement Figure 6: subgroup analysis for E_2_ with different duration treatment. Supplement Figure 7: subgroup analysis for FSH with different duration treatment. Supplement Figure 8: subgroup analysis for LH with different duration treatment. Supplement Figure 9: funnel plot for total effective rate. Supplement Figure 10: funnel plot for E_2_ level. Supplement Figure 11: funnel plot for FSH level. Supplement Figure 12: funnel plot for LH level. Supplement Figure 13: sensitivity analysis for the total effective rate.

## References

[1] Webber L., Davies M., Anderson R. (2016). ESHRE guideline: management of women with premature ovarian insufficiency. *Human Reproduction*.

[2] Chae-Kim J. J., Gavrilova-Jordan L. (2018). Premature ovarian insufficiency: procreative management and preventive strategies. *Biomedicines*.

[3] Lim Y.-M., Jeong K., Lee S. R. (2019). Association between premature ovarian insufficiency, early menopause, socioeconomic status in a nationally representative sample from Korea. *Maturitas*.

[4] Sharif K., Watad A., Bridgewood C., Kanduc D., Amital H., Shoenfeld Y. (2019). Insights into the autoimmune aspect of premature ovarian insufficiency. *Best Practice & Research Clinical Endocrinology & Metabolism*.

[5] Ni Y., Xu D., Lv F. (2019). Prenatal ethanol exposure induces susceptibility to premature ovarian insufficiency. *Journal of Endocrinology*.

[6] Tao X.-Y., Zuo A.-Z., Wang J.-Q., Tao F-B. (2016). Effect of primary ovarian insufficiency and early natural menopause on mortality: a meta-analysis. *Climacteric*.

[7] Podfigurna A., Męczekalski B. (2018). Cardiovascular health in patients with premature ovarian insufficiency. Management of long-term consequences. *Menopausal Review*.

[8] Goh M., Nguyen H. H., Khan N. N., Milat F., Boyle J. A., Vincent A. J. (2019). Identifying and addressing osteoporosis knowledge gaps in women with premature ovarian insufficiency and early menopause: a mixed-methods study. *Clinical Endocrinology*.

[9] Whitcomb B. W., Purdue-Smithe A., Hankinson S. E. (2018). Menstrual cycle characteristics in adolescence and early adulthood are associated with risk of early natural menopause. *The Journal of Clinical Endocrinology & Metabolism*.

[10] Podfigurna-Stopa A., Czyzyk A., Grymowicz M. (2016). Premature ovarian insufficiency: the context of long-term effects. *Journal of Endocrinological Investigation*.

[11] Machura P., Grymowicz M., Rudnicka E. (2018). Premature ovarian insufficiency—hormone replacement therapy and management of long-term consequences. *Menopausal Review*.

[12] Ma K., Wang K. L., Chen Y. X. (2019). Infertility caused by salpingitis treated based on theory of kidney deficiency and blood stasis. *China Journal of Chinese Materia Medica*.

[13] Zhang C. H., Ma K., Yuan B. C. (2019). Bushen Huoxue herbal medicine for treating hyperprolactinemia in women: a meta-analysis. *China Journal of Chinese Materia Medica*.

[14] Shan J., Cheng W., Zhai D. X. (2017). Meta-analysis of Chinese traditional medicine Bushen Huoxue prescription for endometriosis treatment. *Evidence-Based Complementary and Alternative Medicine*.

[15] Jin Z. C., Huang X. T., Yang Y. Q. (2013). Treatment of premature ovarian failure by Bushen Huoxue recipe combined estrogen and progesterone: a clinical research. *Chinese Journal of Integrated Traditional and Western Medicine*.

[16] Xu B. H., Li M. Q., Luo Y. J. (2013). Treatment of premature ovarian failure patients by Bushen Tiaojing recipe combined hormone replacement therapy: a clinical observation. *Chinese Journal of Integrated Traditional and Western Medicine*.

[17] Zeng F. L., Sun W. F., Li J. (2019). Huoxue Zishen recipe treated immunological premature ovarian failure of Shen deficiency blood stasis type: a clinical observation. *Chinese Journal of Integrated Traditional and Western Medicine*.

[18] Shang Y. J., Chen Y., Lu S. (2018). Systematic review and meta-analysis of Bushen Huoxue medicine in treatment of premature ovarian failure. *Chinese Archives of Traditional Chinese Medicine*.

[19] Moher D., Liberati A., Tetzlaff J. (2009). Preferred reporting items for systematic reviews and meta-analyses: the PRISMA statement. *Journal of Clinical Epidemiology*.

[20] Bi D. H. (2015). Observation on the curative effect of traditional Chinese medicine Bushen Tiaochong prescription on ovarian premature aging. *Journal of Practical Gynecologic Endocrinology*.

[21] Fang Q. X., Zou P., Chen D. Q. Clinical observation on treatment of 26 cases of premature ovarian failure with kidney deficiency and blood stasis by Bushen Huoxue decoction.

[22] Gao H., Xia T., Han B. (2007). Clinical study of Bushen Tiaochong recipe on treating premature ovarian failure. *Liaoning Journal of Traditional Chinese Medicine*.

[23] Jiang Y. H., Li X. W., Chen Y. N. (2013). Clinical study on treatment of premature ovarian failure with Yishen Guijing decoction. *China Journal of Pharmaceutical Economics*.

[24] Li Y. Q. (2016). Clinical observation on treatment of premature ovarian failure with traditional Chinese medicine. *Chinese Health Nutrition*.

[25] Ma X. Y. (2015). Clinical analysis of traditional Chinese medicine Bushen Recipe in the treatment of premature ovarian failure. *Journal of New Chinese Medicine*.

[26] Shen H., Hong Y. L., Shi Y. Q. (2013). Clinical observation on 40 cases of premature ovarian failure treated by Zishen Huoxue decoction. *Asia Pacific Traditional Medicine*.

[27] Wang H. M., Zhu X. L., Hu L. S. (2010). Clinical study of Bushen Huoxue method on premature ovarian failure with the immunologic unbalance. *Jiangxi Medical Journal*.

[28] Wang L. P. (2010). Treatment of 48 cases of premature ovarian failure with Bushen recipe. *Shanxi Traditional Chinese Medicine*.

[29] Wang L. X. (2015). Clinical study on treatment of premature ovarian failure with Chinese medicine Bushen Tiao Chong recipe. *Contemporary Medicine*.

[30] Xia Y. Q., Song J. Y., Dong L. (2019). Bushen Huoxue recipe in treatment of premature ovarian failure. *Acta Chinese Medicine*.

[31] Xu B. H., Li M. Q., Luo Y. J. (2014). Effect of Bushen Tiaojing decoction on reproductive axis in patients with premature ovarian failure. *Chinese Journal of Experimental Traditional Medical Formulae*.

[32] Xu B. H., Li M. Q., Zhu Q. F. (2017). Effect of Bushen Tiaojing paste on endocrine and immune regulation of premature ovarian failure patients with Shen deficiency. *Chinese Journal of Integrated Traditional Chinese and Western Medicine*.

[33] Xu B. H., Li M. Q., Xiong S. Y. (2017). Effect of Bushen Tiaojing recipe extract on sexual hormone levels of premature ovarian failure patients with kidney deficiency syndrome. *Journal of Guangzhou University of Traditional Chinese Medicine*.

[34] Xu B. H., Li M. Q., Yu P. (2012). Effect of Bushen Tiaojing decoction on sex hormone of patients with premature ovarian failure. *Journal of Sichuan of Traditional Chinese Medicine*.

[35] Yi W. Y., Gu X. L., Liu C. Q. (2008). Clinical observation of hormone replacement combined with traditional Chinese medicine in the treatment of premature ovarian failure. *Chinese Maternal and Child Health Care*.

[36] Zhao G. X. (2014). Clinical observation on treating 36 cases of premature ovarian failure with Fuchao decoction. *Clinical Journal of Chinese Medicine*.

[37] Zhao X. Y., Xie Y. H., Zhen H. Z. (2019). Curative effect of Bushen Tiaojing cream in treatment of premature ovarian failure (kidney deficiency) and its influnce of sex hormone and immune function. *Chinese Archives of Traditional Chinese Medicine*.

[38] Zhong S. Q. (2019). Clinical study on kidney-nourishing and blood-activating prescription in the treatment of premature ovarian failure of kidney deficiency and blood stasis type. *Chinese Medicine Modern Distance Education of China*.

[39] Zhongwei P., Ye Y. Q., Wang P. J. (2019). Curative effect of Bushen Huoxue decoction on premature ovarian insufficiency of type of kidney deficiency and blood stasis. *Modern Journal of Integrated Traditional Chinese and Western Medicine*.

[40] Sun L. (2019). Phytoestrogens of traditional Chinese medicine for Bushen Huoxue Huayu. *Chinese Journal of Gerontology*.

[41] Zhao L., Zheng H. X., Xu Y. (2017). Research progress in phytoestrogens of traditional Chinese medicine. *China Journal of Chinese Materia Medica*.

[42] Zhang R. H., Shu X. C. (2003). Effect of Chinese herbal recipe extract for replenishing kidney and activating blood circulation (CRKRABC) on osteoblast. *Chinese Journal of Pathophysiology*.

[43] Zhou X. M., Chen Y. Y. (2014). Summary of mechanism of treating diminished ovarian reserve with traditional Chinese medicine of tonifying kidney and activating blood. *Chinese Journal of Traditional Medical Science and Technology*.

[44] Liu H. P., Xiao Y., Li L. (2015). The influencen of Bushen Huoxue prescription on follicular granulosa cell apoptosis of premature ovarian failure (POF) mice. *Chinese Journal of Information on Traditional Chinese Medicine*.

[45] Du J. (2015). Analysis on the compliance of Chinese medicine use in outpatients. *Guide of China Medicine*.

[46] Zhang G. (2012). Compliance assurance for researchers in TCM clinical trials. *Practical Pharmacy and Clinical Remedies*.

[47] Huang B., Jiang M. J. (2015). Progress in clinical research of herbal pastes. *Journal of Practical Traditional Chinese Internal Medicine*.

