# Supplementary material for: Efficacy of Traditional Chinese Medicine Tonifying Kidney (Bushen) and Activating Blood (Huoxue) Prescription for Premature Ovarian Insufficiency: A Systematic Review and Meta-Analysis
Source: Evid Based Complement Alternat Med. 2020 Apr 21;2020:1789304. doi: 10.1155/2020/1789304 (PMC7191427; doi:10.1155/2020/1789304)

Supplement Digital

PubMed retrieval strategy

#1tonifying kidney and activating blood[Title/Abstract]

#2kidney-tonifying and blood-activating[Title/Abstract]

#3huoxue bushen[Title/Abstract]

#4Nourishing Kidney[Title/Abstract]

#5tonifying kidney[Title/Abstract]

#6reinforcing kidney[Title/Abstract]

#7warming kidney[Title/Abstract]

#8warming shen[Title/Abstract]

#9promoting blood [Title/Abstract]

#10activating blood [Title/Abstract]

#11invigorating blood [Title/Abstract]

#12bu shen[Title/Abstract]

#13wen shen[Title/Abstract]

#14zi shen[Title/Abstract]

#15huo xue[Title/Abstract]

#16qu yu[Title/Abstract]

#17hua yu[Title/Abstract]

#18 #l OR to#17

#19Primary Ovarian Insufficiency[MeSH Terms]

#20Menopause, Premature[MeSH Terms]

#21Premature Menopause[Title/Abstract]

#22Premature Ovarian Insufficiency [Title/Abstract]

#23Premature Ovarian Failure[Title/Abstract]

#24Ovarian Failure, Premature[Title/Abstract]

#25Ovarian Insufficiency, Primary[Title/Abstract]

#26decreased ovarian reserve [Title/Abstract]

#27POI[Title/Abstract]

#28POF[Title/Abstract]

#29 #l9 OR to#28

#30randomized controlled trial[Publication Type]

#31controlled clinical trial[Publication Type]

#32randomized[Title/Abstract]

#33randomly[Title/Abstract]

#34placebo[Title/Abstract]

#35 trial[Titlel]

#36 #30 OR to#35

#37#18 AND #29 AND #36

Supplement Table 1: Composition of prescription in the included studies.

References	Prescription	Composition of prescription
Jin et al. 2013	TKABP	Prepared Radix Rehmanniae 15 g, Dodder 15 g, Antler Glue 15 g, Glossy Privet Fruit 15 g, Psoralen 10 g, Cornus 10 g, White Peony 10 g, Angelica 10 g, Rhizome of Chuanxiong 10 g, Peach Kernel 10 g, Safflower 10 g
Xu et al. 2013	TKABP	Prepared Radix Rehmanniae 20 g, Dodder 15 g, Chinese Yam 15 g, Cornus Officinalis 10 g, Lycium barbarum 15 g, Polygonatum Sibiricum 15 g, Motherwort 15 g, Herba Lycopi 10 g, Radix Cyathulae 15 g, Angelica 10 g, Liquorice Root 3 g
Bi 2015	TKABP	Prepared Radix Rehmanniae 15 g, Dodder 15 g, Polygonatum Sibiricum 15 g, Fluoritum 15 g, Morinda 10 g, Cistanche 10 g, Schisandra Chinensis 6 g, Angelica 10 g, Rhizome of Chuanxiong 6 g
Zeng et al. 2019	TKABP	Prepared Radix Rehmanniae 15 g, Dodder 10 g, Eclipta 10 g, Glossy Privet Fruit 10 g, Dangshen 10 g, White Peony 10 g, Cistanche 10 g, Caulis Spatholobi 15 g, Angelica 10 g, Aerial Parts of Epimedium 10 g, Polygonum Multiflorum 10 g, Liquorice Root 10 g
Fang et al. 2015	TKABP	Prepared Radix Rehmanniae 20 g, Dodder 30 g, Dipsacus 20 g, Angelica 12 g, Glossy Privet Fruit 15 g, Poria Cocos 20 g, White Atractylodes Rhizome 15 g, Spatholobi 30 g, Radix Cyathulae 12 g, Rhizome of Chuanxiong 12 g, Herba Lycopi 12g, Liquorice Root 6 g
Gao et al. 2007	TKABP	Prepared Radix Rehmanniae 15 g, Dodder 15 g, Polygonatum Sibiricum15 g, Schisandra Chinensis 6 g, Morinda 10 g, Cistanche 10 g, Fluoritum 15 g, Rhizome of Chuanxiong 6 g, Angelica 10 g
Jiang et al.2013	TKABP	Prepared Radix Rehmanniae 20 g, Dodder 20 g, Chinese Yam 30 g, Cornus Officinalis 20 g, Polygonum Multiflorum 15 g, Morinda 15 g, Aerial Parts of Epimedium 15 g, Cyperus Rotundus15 g, Eucommia 20 g, Polygonatum Sibiricum15 g, Lycium Barbarum 15 g, Angelica 20 g, Caulis Spatholobi 20 g, Motherwort 25 g, Antler Glue 10 g
Li 2016	TKABP	Prepared Radix Rehmanniae 15 g, Dodder 15 g, Glossy Privet Fruit 15 g, Antler Glue 15 g, Cornus Officinalis 10 g, Angelica 10 g, White Peony 10 g, Rhizome of Chuanxiong 10 g, Peach Kernel 10 g, Safflower 10 g
Ma 2015	TKABP	Prepared Radix Rehmanniae 15 g, Dodder 15 g, Astragalus 15 g, Polygonum Multiflorum15 g, Lycium Barbarum 10 g, Placenta Hominis 10 g, Angelica 10 g, Rhizome of Chuanxiong 6 g, Red Dates 5 g
Shen et al. 2013	TKABP	Prepared Radix Rehmanniae 10 g, Dodder 10 g, Turtle Shell 10 g, Polygonum Multiflorum10 g, Cornus Officinalis 10 g, Chinese Yam 10 g, Lycium Barbarum 10 g, Dipsacus 10 g, Poria Cocos 10 g, Radix Pseudostellariae10 g, Salvia 15 g
Wang et al. 2010	TKABP	Prepared Radix Rehmanniae 15g, Dodder 10 g, Chinese Yam 20 g, Radix Cyathulae 10 g, Lycium barbarum 10 g, Angelica 15 g,Salvia 20 g, Polygonatum Sibiricum10 g
Wang 2010	TKABP	Prepared Radix Rehmanniae 10 g, Chinese Yam 20 g, Dodder 12 g, Glossy Privet Fruit 15 g, Aerial Parts of Epimedium 10 g, Tortoise Plastron Glue 10 g, Antler Glue 10 g, Cornus Officinalis 15 g, Eucommia 13 g + Dahuang Zhechong Pill
Wang 2015	TKABP	Prepared Radix Rehmanniae 20 g, Chinese Yam 20 g, Dodder 10 g, Glossy Privet Fruit 10 g, Lycium barbarum 15 g, Salvia 15 g, Eclipta 10 g, Placenta Hominis 12 g, Red Dates 10 g
Xia et al. 2019	TKABP	Prepared Radix Rehmanniae 12 g, Dodder 12 g, Morinda 12 g, Placenta Hominis 3 g, Aerial Parts of Epimedium 15 g, Raspberry 12 g, Astragalus 20 g, Salvia 20 g, Angelica 20 g, Dangshen 20 g
Xu et al. 2014	TKABP	Prepared Radix Rehmanniae 20 g, Dodder 15 g, Chinese Yam 15 g, Cornus Officinalis 10 g, Lycium barbarum 15 g, Motherwort 15 g, Radix Cyathulae 15 g, Dipsacus 15 g, Angelica 10 g, Poria Cocos 10 g, Tree Peony Bark 10 g
Xu et al. 2017	TKABP	Prepared Radix Rehmanniae 15 g, Dodder 12 g, Chinese Yam 15 g, Cornus Officinalis 10 g, Lycium barbarum 12 g, Motherwort 10 g, Radix Cyathulae 10 g, Dipsacus 10 g, Poria Cocos 10 g, Tree Peony Bark 10 g, Mulberry 10 g
Xu et al. 2017*	TKABP	Prepared Radix Rehmanniae 15 g, Dodder 12 g, Chinese Yam 15 g, Cornus Officinalis 10 g, Lycium barbarum 12 g, Motherwort 10 g, Radix Cyathulae 10 g, Dipsacus 10 g, Poria Cocos 10 g, Tree Peony Bark 10 g, Mulberry 10 g
Xu et al. 2012	TKABP	Prepared Radix Rehmanniae 20 g, Dodder 15 g, Chinese Yam 15 g, Cornus Officinalis 10 g, Lycium barbarum 15 g, Curculigo 10 g, Aerial Parts of Epimedium 10 g, Motherwort 15 g, Radix Cyathulae 15 g, Dipsacus 10 g, Poria Cocos 12 g, Herba Lycopi 10 g, Angelica 10 g, Liquorice Root 3 g
Yi et al. 2008	TKABP	Prepared Radix Rehmanniae 20 g, Chinese Yam 20 g, Polygonum Multiflorum 15 g, Placenta Hominis 15 g, Eclipta 15 g, Glossy Privet Fruit 12 g, Tree Peony Bark 12 g, Red Peony Root 12 g, Peach Kernel 10 g, Safflower 6 g, Liquorice Root 6 g
Zhao 2014	TKABP	Prepared Radix Rehmanniae 15 g, Dodder 15 g, Glossy Privet Fruit 15 g, Polygonum Multiflorum 15 g, Cornus Officinalis 10 g, White Peony 15 g, Radix Cyathulae 10 g, Lycium barbarum 15 g, Dipsacus 10 g, Placenta Hominis 10 g, Angelica 15 g, Salvia 15 g
Zhao et al. 2019	TKABP	Prepared Radix Rehmanniae 15 g, Dodder 12 g, Chinese Yam 15 g, Glossy Privet Fruit 9 g, Polygonum Multiflorum 12 g, Cornus Officinalis 10 g, Lycium barbarum 12 g, Motherwort 10 g, Radix Cyathulae 10 g, Dipsacus 10 g, Angelica 9 g, Poria Cocos 10 g, Tree Peony Bark 10 g, Mulberry 10 g, Salvia 9 g, Cistanche 12 g, Eclipta 9 g
Zhong 2019	TKABP	Prepared Radix Rehmanniae 15 g, Chinese Yam 15 g, Placenta Hominis10g, Radix Cyathulae 12 g, Angelica 15 g, Eucommia 10g, Peach Kernel 15 g, Safflower 9 g, Cornus Officinalis 10 g, Salvia 10 g, Motherwort 20 g, Liquorice Root 6 g
Zhong et al. 2019	TKABP	Prepared Radix Rehmanniae 12 g, Dodder 10 g, Chinese Yam 10 g, Cornus Officinalis10 g, Lycium barbarum 10 g, White Peony 10 g, Angelica 10 g, Rhizome of Chuanxiong 6 g, Cyperi Rhizoma 10g, Salvia 10 g, Radix Cyathulae 10 g, Liquorice Root 6 g

Supplement Table2: PRISMA checklist.

Section/Topic	#	Checklist Item	Reported on Page #
**TITLE**			
Title	1	Identify the report as a systematic review and meta-analysis	#1
**ABSTRACT**			
Structured summary	2	Provide a structured summary including, as applicable: background; objectives; data sources; study eligibility criteria, participants, and interventions; study appraisal and synthesis methods; results; limitations; conclusions and implications of key findings; systematic review registration number.	#2
**INTRODUCTION**			

Rationale	3	Describe the rationale for the review in the context of what is already known.	#3
Objectives	4	Provide an explicit statement of questions being addressed with reference to participants, interventions, comparisons, outcomes, and study design (PICOS).	#3
**METHODS**			
Protocol and registration	5	Indicate if a review protocol exists, if and where it can be accessed (e.g., Web address), and, if available, provide registration information including registration number.	#3
Eligibility criteria	6	Specify study characteristics (e.g., PICOS, length of follow-up) and report characteristics (e.g., years considered, language, publication status) used as criteria for eligibility, giving rationale.	#3-4
Information sources	7	Describe all information sources (e.g., databases with dates of coverage, contact with study authors to identify additional studies) in the search and date last searched.	#3-4
Search	8	Present full electronic search strategy for at least one database, including any limits used, such that it could be repeated.	#4
Study selection	9	State the process for selecting studies (i.e., screening, eligibility, included in systematic review, and, if applicable, included in the meta-analysis).	#4-5
Data collection process	10	Describe method of data extraction from reports (e.g., piloted forms, independently, in duplicate) and any processes for obtaining and confirming data from investigators.	#5
Data items	11	List and define all variables for which data were sought (e.g., PICOS, funding sources) and any assumptions and simplifications made.	#4-5
Risk of bias in individual studies	12	Describe methods used for assessing risk of bias of individual studies (including specification of whether this was done at the study or outcome level), and how this information is to be used in any data synthesis.	#5
Summary measures	13	State the principal summary measures (e.g., risk ratio, difference in means).	#5
Synthesis of results	14	Describe the methods of handling data and combining results of studies, if done, including measures of consistency (e.g., I^2) for each meta-analysis.	#5
Section/Topic	#	Checklist Item	Reported on Page #
Risk of bias across studies	15	Specify any assessment of risk of bias that may affect the cumulative evidence (e.g., publication bias, selective reporting within studies).	#5
Additional analyses	16	Describe methods of additional analyses (e.g., sensitivity or subgroup analyses, meta-regression), if done, indicating which were pre-specified.	#5
**RESULTS**			
Study selection	17	Give numbers of studies screened, assessed for eligibility, and included in the review, with reasons for exclusions at each stage, ideally with a flow diagram.	#5-6
Study characteristics	18	For each study, present characteristics for which data were extracted (e.g., study size, PICOS, follow-up period) and provide the citations.	#6
Risk of bias within studies	19	Present data on risk of bias of each study and, if available, any outcome level assessment (see item 12).	#6
Results of individual studies	20	For all outcomes considered (benefits or harms), present, for each study: (a) simple summary data for each intervention group (b) effect estimates and confidence intervals, ideally with a forest plot.	#6-7
Synthesis of results	21	Present the main results of the review. If meta-analyses done, include for each, confidence intervals and measures of consistency.	#6-7
Risk of bias across studies	22	Present results of any assessment of risk of bias across studies (see Item 15).	#7
Additional analysis	23	Give results of additional analyses, if done (e.g., sensitivity or subgroup analyses, meta-regression [see Item 16]).	#7
**DISCUSSION**			
Summary of evidence	24	Summarize the main findings including the strength of evidence for each main outcome; consider their relevance to key groups (e.g., healthcare providers, users, and policy makers).	#8
Limitations	25	Discuss limitations at study and outcome level (e.g., risk of bias), and at review-level (e.g., incomplete retrieval of identified research, reporting bias).	#9
Conclusions	26	Provide a general interpretation of the results in the context of other evidence, and implications for future research.	#9
**FUNDING**			
Funding	27	Describe sources of funding for the systematic review and other support (e.g., supply of data); role of funders for the systematic review.	#1

Supplement Figure 1. Subgroup analysis for total effective rate with different dosage forms.

Study or Subgroup	TKABP Events	TKABP Total	Control Events	Control Total	Weight	Risk Ratio M-H, Random, 95% CI
**3.8.1 Herbal decoction**						
Bi2015	48	50	46	50	12.6%	1.04 [0.94, 1.15]
Fang2015	23	26	20	26	3.8%	1.15 [0.89, 1.48]
Gao2007	28	30	27	30	7.9%	1.04 [0.89, 1.21]
Jiang2013	41	45	37	45	7.3%	1.11 [0.94, 1.31]
Jin2013	80	86	81	88	14.4%	1.01 [0.93, 1.10]
Li2016	32	34	27	34	5.8%	1.19 [0.98, 1.43]
Ma2015	44	50	34	50	4.8%	1.29 [1.04, 1.61]
Shen2013	43	68	29	60	2.5%	1.31 [0.95, 1.80]
Wang2010	17	20	18	20	4.2%	0.94 [0.75, 1.19]
Wang2015	47	50	45	50	10.9%	1.04 [0.93, 1.17]
Wangli2010	34	48	28	42	3.1%	1.06 [0.80, 1.41]
Xu2012	21	23	16	23	2.8%	1.31 [0.97, 1.77]
Xu2013	17	24	16	24	1.8%	1.06 [0.73, 1.56]
Xu2014	25	30	21	30	3.1%	1.19 [0.90, 1.58]
Xub(1)2017	17	23	12	23	1.3%	1.42 [0.89, 2.25]
Yi2008	6	22	13	20	0.5%	0.42 [0.20, 0.89]
Zhao2014	29	36	20	24	4.1%	0.97 [0.76, 1.23]
Zhong2019	33	38	25	38	3.6%	1.32 [1.02, 1.71]
Zhongwei2019	20	20	17	20	5.3%	1.17 [0.96, 1.43]
**Subtotal (95% CI)**		**723**		**697**	**100.0%**	**1.09 [1.03, 1.15]**
Total events	605		532			
Heterogeneity: Tau² = 0.00; Chi² = 24.77, df = 18 (P = 0.13); I² = 27%						
Test for overall effect: Z = 3.19 (P = 0.001)						
**3.8.2 Herbal paste**						
Xu2017	27	34	21	34	66.8%	1.29 [0.94, 1.76]
Xub(2)2017	18	23	12	23	33.2%	1.50 [0.96, 2.34]
**Subtotal (95% CI)**		**57**		**57**	**100.0%**	**1.35 [1.05, 1.75]**
Total events	45		33			
Heterogeneity: Tau² = 0.00; Chi² = 0.31, df = 1 (P = 0.58); I² = 0%						
Test for overall effect: Z = 2.30 (P = 0.02)						

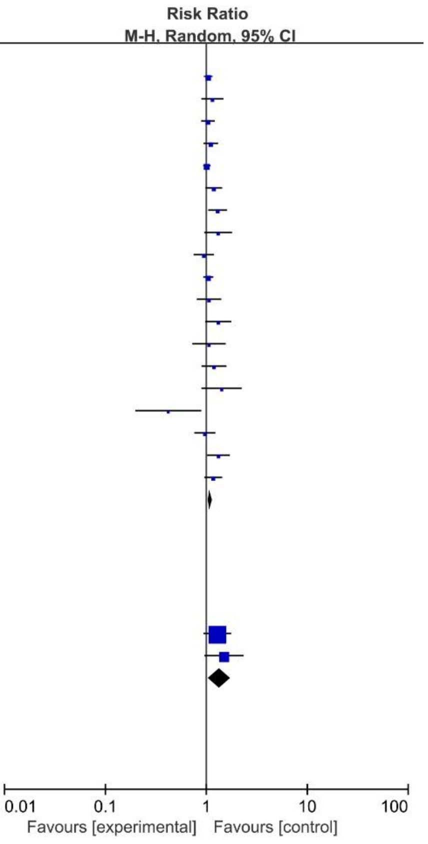

Supplement Figure 2. Subgroup analysis for total effective rate with different duration treatment.

Study or Subgroup	TKABP Events	TKABP Total	Control Events	Control Total	Weight	Risk Ratio M-H, Random, 95% CI
**2.1.1 3M**						
Fang2015	23	26	20	26	6.3%	1.15 [0.89, 1.48]
Gao2007	28	30	27	30	13.1%	1.04 [0.89, 1.21]
Jiang2013	41	45	37	45	12.0%	1.11 [0.94, 1.31]
Ma2015	44	50	34	50	8.1%	1.29 [1.04, 1.61]
Wang2015	47	50	45	50	17.8%	1.04 [0.93, 1.17]
Wangli2010	34	48	28	42	5.3%	1.06 [0.80, 1.41]
Xu2012	21	23	16	23	4.7%	1.31 [0.97, 1.77]
Xu2013	17	24	16	24	3.1%	1.06 [0.73, 1.56]
Xu2014	25	30	21	30	5.2%	1.19 [0.90, 1.58]
Xu2017	27	34	21	34	4.3%	1.29 [0.94, 1.76]
Xub(1)2017	17	23	12	23	2.2%	1.42 [0.89, 2.25]
Xub(2)2017	18	23	12	23	2.3%	1.50 [0.96, 2.34]
Yi2008	6	22	13	20	0.8%	0.42 [0.20, 0.89]
Zhong2019	33	38	25	38	6.0%	1.32 [1.02, 1.71]
Zhongwei2019	20	20	17	20	8.9%	1.17 [0.96, 1.43]
**Subtotal (95% CI)**		**486**		**478**	**100.0%**	**1.14 [1.06, 1.22]**
Total events	401		344			
Heterogeneity: Tau² = 0.00; Chi² = 17.62, df = 14 (P = 0.22); I² = 21%						
Test for overall effect: Z = 3.71 (P = 0.0002)						
**2.1.2 ＞3M**						
Bi2015	48	50	46	50	30.8%	1.04 [0.94, 1.15]
Jin2013	80	86	81	88	37.2%	1.01 [0.93, 1.10]
Li2016	32	34	27	34	11.6%	1.19 [0.98, 1.43]
Shen2013	43	68	29	60	4.6%	1.31 [0.95, 1.80]
Wang2010	17	20	18	20	8.1%	0.94 [0.75, 1.19]
Zhao2014	29	36	20	24	7.7%	0.97 [0.76, 1.23]
**Subtotal (95% CI)**		**294**		**276**	**100.0%**	**1.04 [0.97, 1.12]**
Total events	249		221			
Heterogeneity: Tau² = 0.00; Chi² = 6.33, df = 5 (P = 0.28); I² = 21%						
Test for overall effect: Z = 1.17 (P = 0.24)						

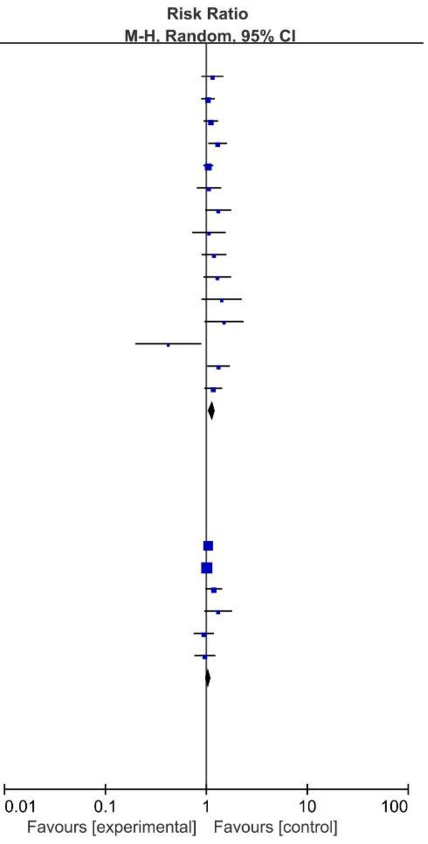

Supplement Figure 3. Subgroup analysis for E_2 with different dosage forms.

Study or Subgroup	TKABP Mean	TKABP SD	TKABP Total	Control Mean	Control SD	Control Total	Weight	Std. Mean Difference IV, Random, 95% CI
**3.5.1 Herbal decoction**								
Fang2015	45.87	10.35	26	33.55	8.58	26	5.8%	1.28 [0.68, 1.88]
Gao2007	63	7.39	30	60	5.33	30	6.0%	0.46 [-0.05, 0.97]
Jin2013	33.55	3.76	86	35.55	3.87	88	6.2%	-0.52 [-0.82, -0.22]
Li2016	13.45	2.72	34	19.34	3.71	34	5.9%	-1.79 [-2.36, -1.22]
Shen2013	55.04	6.28	68	48.02	5.89	60	6.2%	1.14 [0.77, 1.52]
Wang2010	95.28	15.76	20	77.36	12.67	20	5.7%	1.23 [0.55, 1.91]
Wangli2010	110	13.45	48	113	12.35	42	6.1%	-0.23 [-0.65, 0.19]
Xia2019	26.57	20.67	30	31.93	15.01	30	6.0%	-0.29 [-0.80, 0.22]
Xu2012	93.4	9.7	23	92.9	9.6	23	5.9%	0.05 [-0.53, 0.63]
Xu2013	75.4	7.6	24	80.1	8.3	24	5.9%	-0.58 [-1.16, -0.00]
Xu2014	73.4	7.5	30	81.2	8.4	30	5.9%	-0.97 [-1.50, -0.43]
Xub(1)2017	71.96	5.33	23	48.43	5.44	23	4.9%	4.29 [3.21, 5.38]
Yi2008	16	4.8	22	26.7	12.8	20	5.8%	-1.11 [-1.76, -0.45]
Zeng2019	58.68	13.72	32	46.03	14.77	31	6.0%	0.88 [0.36, 1.40]
Zhao2014	93.21	22.58	36	95.01	20.5	24	6.0%	-0.08 [-0.60, 0.44]
Zhong2019	132.46	11.32	38	112.69	12.45	38	6.0%	1.64 [1.12, 2.17]
Zhongwei2019	107.36	18.83	20	104.22	12.78	20	5.8%	0.19 [-0.43, 0.81]
**Subtotal (95% CI)**			**590**			**563**	**100.0%**	**0.29 [-0.21, 0.79]**
Heterogeneity: Tau² = 1.02; Chi² = 254.00, df = 16 (P < 0.00001); I² = 94%								
Test for overall effect: Z = 1.13 (P = 0.26)								
**3.5.2 Herbal paste**								
Xu2017	71.12	7.99	34	49.21	4.94	34	33.8%	3.26 [2.52, 4.00]
Xub(2)2017	72.58	7.88	23	48.43	5.44	23	26.8%	3.51 [2.56, 4.45]
Zhao2019	836.45	58.15	39	693.66	58.14	39	39.4%	2.43 [1.84, 3.02]
**Subtotal (95% CI)**			**96**			**96**	**100.0%**	**3.00 [2.32, 3.67]**
Heterogeneity: Tau² = 0.21; Chi² = 4.90, df = 2 (P = 0.09); I² = 59%								
Test for overall effect: Z = 8.71 (P < 0.00001)								

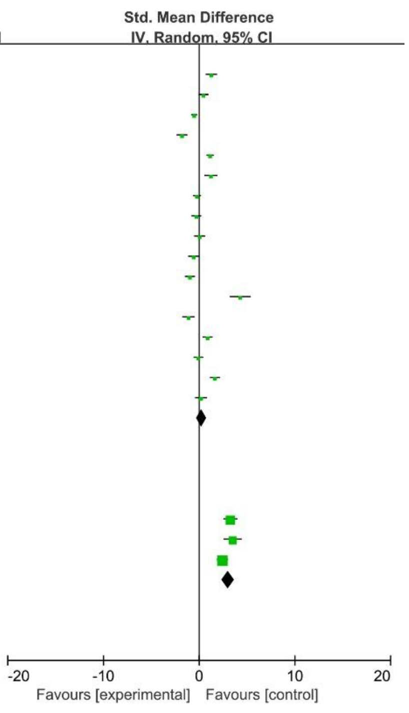

Supplement Figure 4. Subgroup analysis for FSH with different dosage forms.

Study or Subgroup	TKABP Mean	TKABP SD	TKABP Total	Control Mean	Control SD	Control Total	Weight	Std. Mean Difference IV, Random, 95% CI
**3.6.1 Herbal decoction**								
Fang2015	24.34	9.57	26	41.63	12.57	26	5.6%	-1.52 [-2.15, -0.90]
Gao2007	30.87	5.34	30	34.25	4.68	30	5.9%	-0.66 [-1.19, -0.14]
Jin2013	20.12	4.21	86	18.56	5.22	88	6.5%	0.33 [0.03, 0.63]
Li2016	23.07	3.17	34	35.64	5.81	34	5.5%	-2.66 [-3.32, -1.99]
Shen2013	22.02	10.09	68	31.82	13.32	60	6.3%	-0.83 [-1.19, -0.47]
Wang2010	40.52	11.28	20	41.56	16.85	20	5.6%	-0.07 [-0.69, 0.55]
Wangli2010	40.39	10.65	48	40.43	17.65	42	6.2%	-0.00 [-0.42, 0.41]
Xia2019	60.66	20.51	30	67.78	28.19	30	6.0%	-0.29 [-0.79, 0.22]
Xu2012	28.3	5.4	23	27.8	5.5	23	5.8%	0.09 [-0.49, 0.67]
Xu2013	30.6	4.9	24	29.8	5.2	24	5.8%	0.16 [-0.41, 0.72]
Xu2014	41.5	4.8	30	39.8	5.1	30	6.0%	0.34 [-0.17, 0.85]
Xub(1)2017	40.75	5.39	23	46.88	5.32	23	5.6%	-1.13 [-1.75, -0.50]
Yi2008	64	20.6	22	45.7	23.1	20	5.6%	0.82 [0.19, 1.46]
Zeng2019	49.32	11.75	32	56.17	14.33	31	6.0%	-0.52 [-1.02, -0.01]
Zhao2014	28.17	15.02	36	30.48	14.74	24	5.9%	-0.15 [-0.67, 0.36]
Zhong2019	17.28	3.49	38	22.49	4.36	38	6.0%	-1.31 [-1.80, -0.81]
Zhongwei2019	13.29	4.99	20	16.33	6.09	20	5.6%	-0.54 [-1.17, 0.10]
**Subtotal (95% CI)**			**590**			**563**	**100.0%**	**-0.46 [-0.82, -0.09]**
Heterogeneity: Tau² = 0.50; Chi² = 138.57, df = 16 (P < 0.00001); I² = 88%								
Test for overall effect: Z = 2.47 (P = 0.01)								
**3.6.2 Herbal paste**								
Xu2017	42.86	5.86	34	46.26	6.21	34	36.5%	-0.56 [-1.04, -0.07]
Xub(2)2017	42.38	5.18	23	46.88	5.32	23	23.5%	-0.84 [-1.45, -0.24]
Zhao2019	11.78	8.32	39	18.75	8.35	39	40.0%	-0.83 [-1.29, -0.36]
**Subtotal (95% CI)**			**96**			**96**	**100.0%**	**-0.73 [-1.03, -0.44]**
Heterogeneity: Tau² = 0.00; Chi² = 0.79, df = 2 (P = 0.67); I² = 0%								
Test for overall effect: Z = 4.90 (P < 0.00001)								

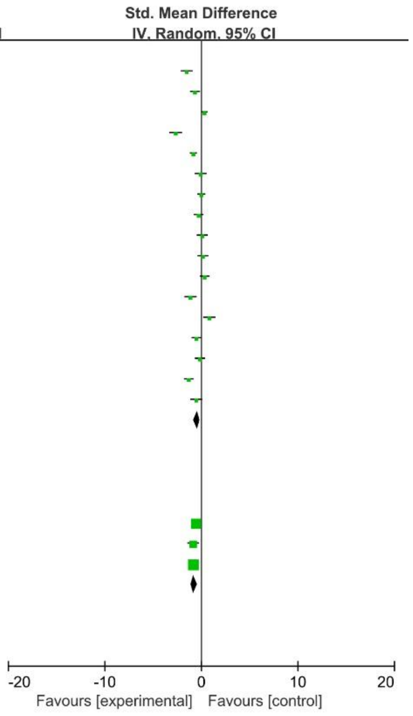

Supplement Figure 5. Subgroup analysis for LH with different dosage forms.

Study or Subgroup	TKABP Mean	TKABP SD	TKABP Total	Control Mean	Control SD	Control Total	Weight	Std. Mean Difference IV, Random, 95% CI
**3.7.1 Herbal decoction**								
Fang2015	13.78	3.46	26	22.54	6.72	26	6.4%	-1.61 [-2.25, -0.98]
Gao2007	23.23	5.01	30	21.41	6.87	30	6.7%	0.30 [-0.21, 0.81]
Jin2013	15.32	2.1	86	14.11	2.34	88	7.2%	0.54 [0.24, 0.84]
Li2016	33.21	5.7	34	25.89	3.02	34	6.6%	1.59 [1.04, 2.14]
Shen2013	17.53	8.26	68	18.03	9.36	60	7.2%	-0.06 [-0.40, 0.29]
Wang2010	30.47	16.85	20	31.25	11.86	20	6.4%	-0.05 [-0.67, 0.57]
Wangli2010	37.95	12.38	48	42.76	9.56	42	7.0%	-0.43 [-0.85, -0.01]
Xu2012	27.1	4.3	23	26.8	4.5	23	6.5%	0.07 [-0.51, 0.65]
Xu2013	29.1	4.3	24	28.2	4.5	24	6.6%	0.20 [-0.37, 0.77]
Xu2014	29.8	3.9	30	28.3	3.8	30	6.7%	0.38 [-0.13, 0.90]
Xub(1)2017	28.38	4.04	23	31.21	4.33	23	6.5%	-0.66 [-1.26, -0.07]
Zeng2019	20.01	8.23	32	28.08	7.98	31	6.7%	-0.98 [-1.51, -0.46]
Zhao2014	12.55	2.37	38	17.31	3.16	38	6.7%	-1.69 [-2.21, -1.16]
Zhong2019	6.48	2.22	20	7.09	2.47	20	6.4%	-0.25 [-0.88, 0.37]
Zhongwei2019	6.48	2.22	20	7.09	2.47	20	6.4%	-0.25 [-0.88, 0.37]
**Subtotal (95% CI)**			**522**			**509**	**100.0%**	**-0.19 [-0.59, 0.21]**
Heterogeneity: Tau² = 0.55; Chi² = 134.73, df = 14 (P < 0.00001); I² = 90%								
Test for overall effect: Z = 0.91 (P = 0.36)								
**3.7.2 Herbal paste**								
Xu2017	28.08	3.45	34	31.09	4.15	34	36.1%	-0.78 [-1.27, -0.29]
Xub(2)2017	28.79	4.09	23	31.21	4.33	23	25.3%	-0.56 [-1.16, 0.03]
Zhao2019	15.51	8.89	39	25.37	8.93	39	38.6%	-1.10 [-1.57, -0.62]
**Subtotal (95% CI)**			**96**			**96**	**100.0%**	**-0.85 [-1.14, -0.55]**
Heterogeneity: Tau² = 0.00; Chi² = 1.99, df = 2 (P = 0.37); I² = 0%								
Test for overall effect: Z = 5.59 (P < 0.00001)								

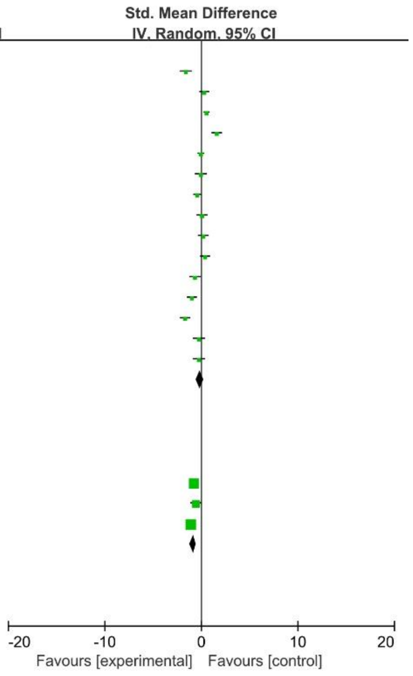

Supplement Figure 6. Subgroup analysis for E_2 with different duration treatment.

Study or Subgroup	TKABP Mean	TKABP SD	TKABP Total	Control Mean	Control SD	Control Total	Weight	Std. Mean Difference IV, Random, 95% CI
**2.2.1 3M**								
Fang2015	45.87	10.35	26	33.55	8.58	26	7.8%	1.28 [0.68, 1.88]
Gao2007	63	7.39	30	60	5.33	30	7.9%	0.46 [-0.05, 0.97]
Wangli2010	110	13.45	48	113	12.35	42	8.0%	-0.23 [-0.65, 0.19]
Xu2012	93.4	9.7	23	92.9	9.6	23	7.8%	0.05 [-0.53, 0.63]
Xu2013	75.4	7.6	24	80.1	8.3	24	7.8%	-0.58 [-1.16, -0.00]
Xu2017	71.12	7.99	34	49.21	4.94	34	7.6%	3.26 [2.52, 4.00]
Xub(1)2017	71.96	5.33	23	48.43	5.44	23	7.0%	4.29 [3.21, 5.38]
Xub(2)2017	72.58	7.88	23	48.43	5.44	23	7.2%	3.51 [2.56, 4.45]
Yi2008	16	4.8	22	26.7	12.8	20	7.7%	-1.11 [-1.76, -0.45]
Zeng2019	58.68	13.72	32	46.03	14.77	31	7.9%	0.88 [0.36, 1.40]
Zhao2019	836.45	58.15	39	693.66	58.14	39	7.8%	2.43 [1.84, 3.02]
Zhong2019	132.46	11.32	38	112.69	12.45	38	7.9%	1.64 [1.12, 2.17]
Zhongwei2019	107.36	18.83	20	104.22	12.78	20	7.7%	0.19 [-0.43, 0.81]
**Subtotal (95% CI)**			**382**			**373**	**100.0%**	**1.19 [0.44, 1.94]**
Heterogeneity: Tau² = 1.79; Chi² = 245.57, df = 12 (P < 0.00001); I² = 95%								
Test for overall effect: Z = 3.12 (P = 0.002)								
**2.2.2 ＞3M**								
Jin2013	33.55	3.76	86	35.55	3.87	88	14.9%	-0.52 [-0.82, -0.22]
Li2016	13.45	2.72	34	19.34	3.71	34	14.1%	-1.79 [-2.36, -1.22]
Shen2013	55.04	6.28	68	48.02	5.89	60	14.7%	1.14 [0.77, 1.52]
Wang2010	95.28	15.76	20	77.36	12.67	20	13.6%	1.23 [0.55, 1.91]
Xia2019	26.57	20.67	30	31.93	15.01	30	14.3%	-0.29 [-0.80, 0.22]
Xu2014	73.4	7.5	30	81.2	8.4	30	14.2%	-0.97 [-1.50, -0.43]
Zhao2014	93.21	22.58	36	95.01	20.5	24	14.3%	-0.08 [-0.60, 0.44]
**Subtotal (95% CI)**			**304**			**286**	**100.0%**	**-0.18 [-0.94, 0.57]**
Heterogeneity: Tau² = 0.96; Chi² = 108.55, df = 6 (P < 0.00001); I² = 94%								
Test for overall effect: Z = 0.48 (P = 0.63)								

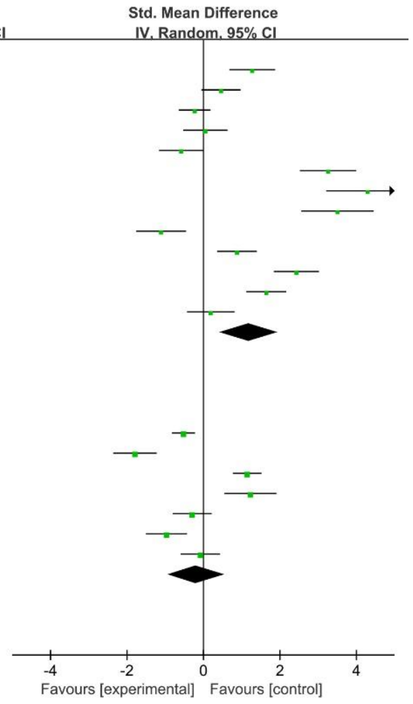

Supplement Figure 7. Subgroup analysis for FSH with different duration treatment.

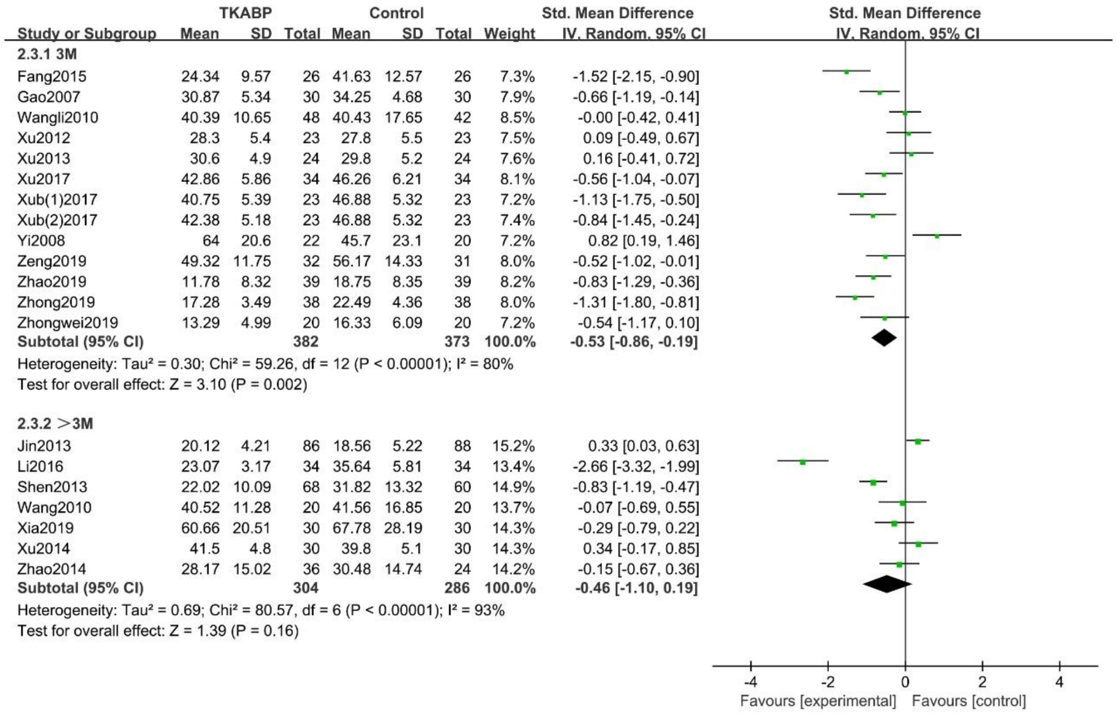

Supplement Figure 8. Subgroup analysis for LH with different duration treatment.

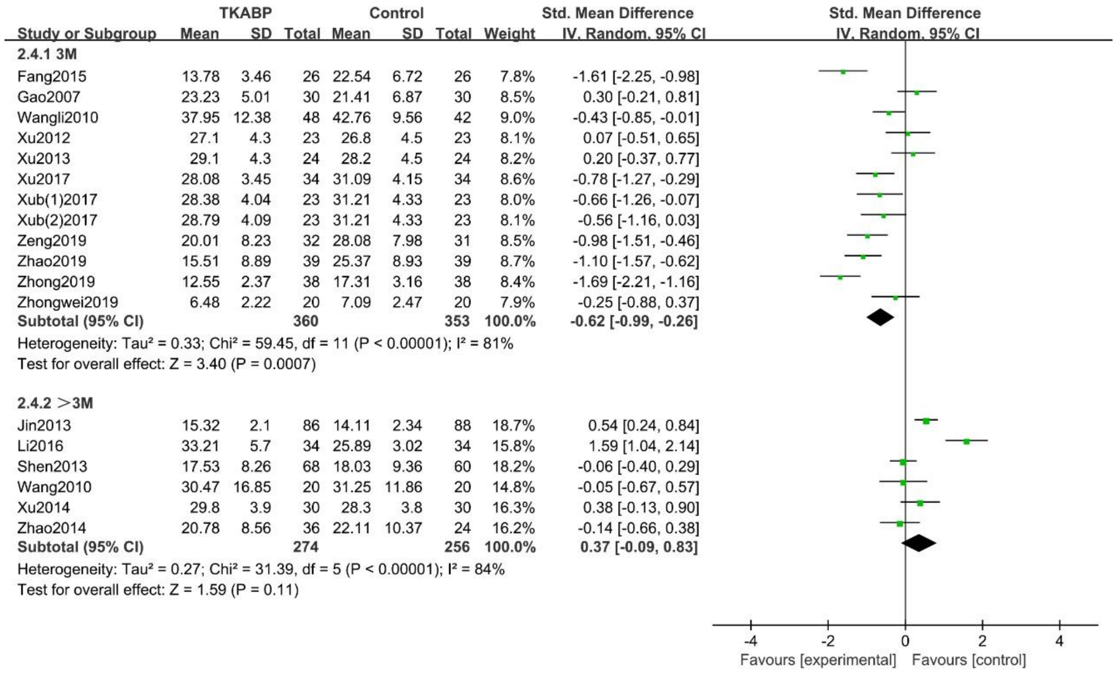

Supplement Figure 9. Funnel plot for total effective rate.

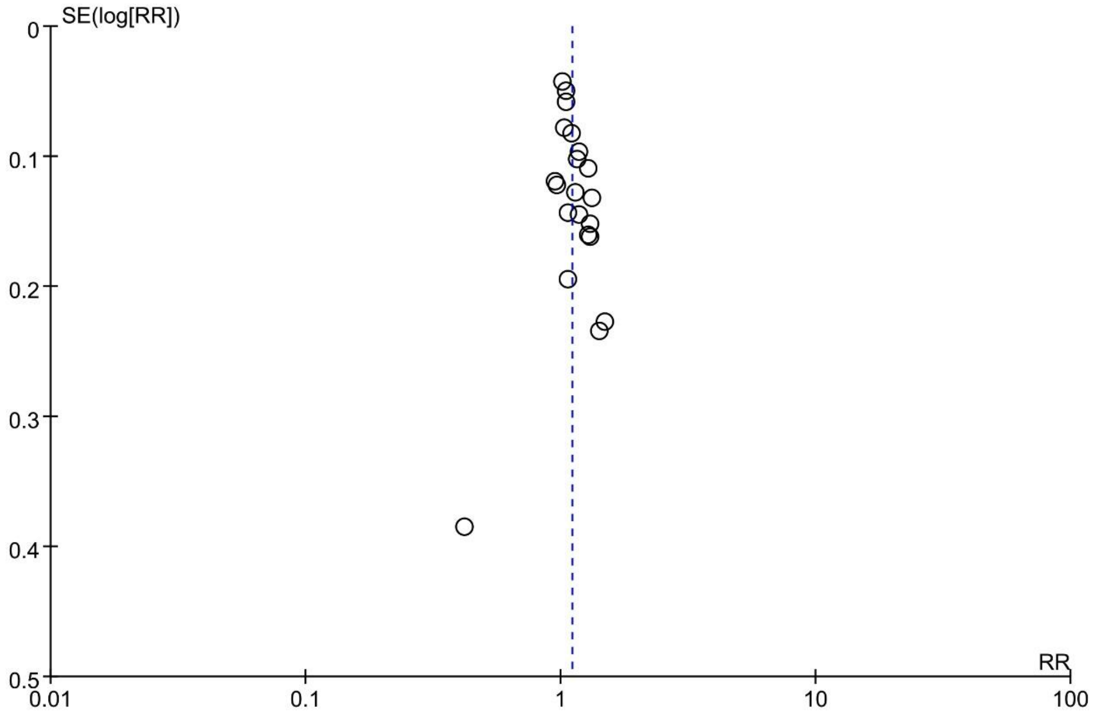

Supplement Figure 10. Funnel plot for E_2 level.

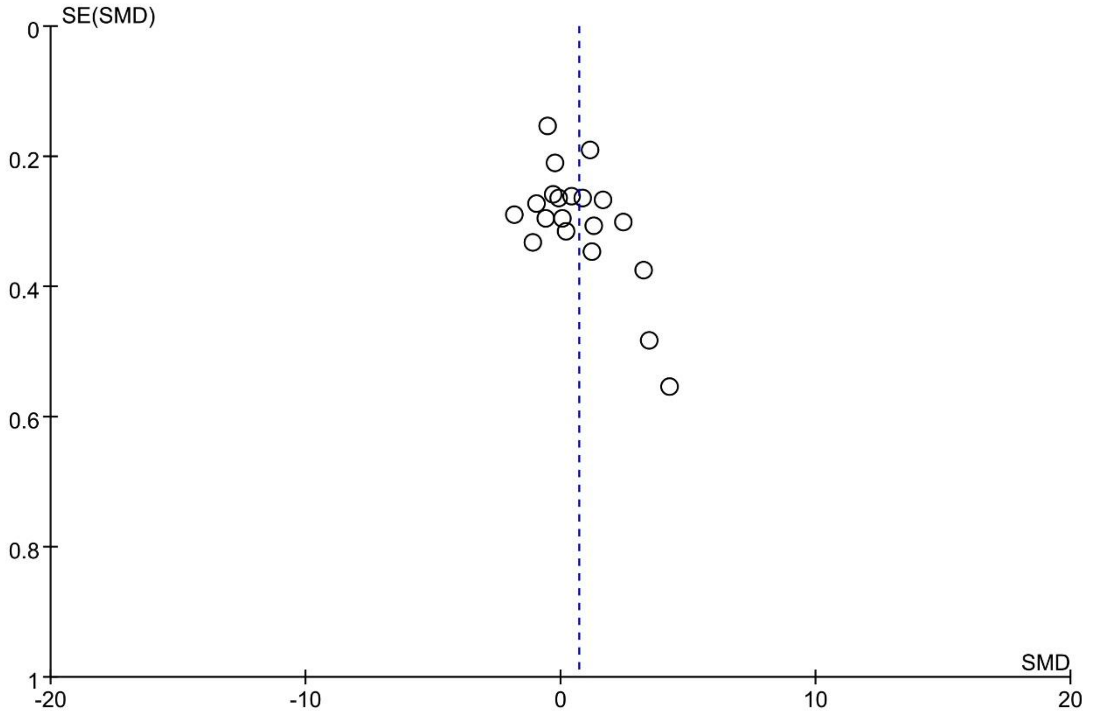

Supplement Figure 11. Funnel plot for FSH level.

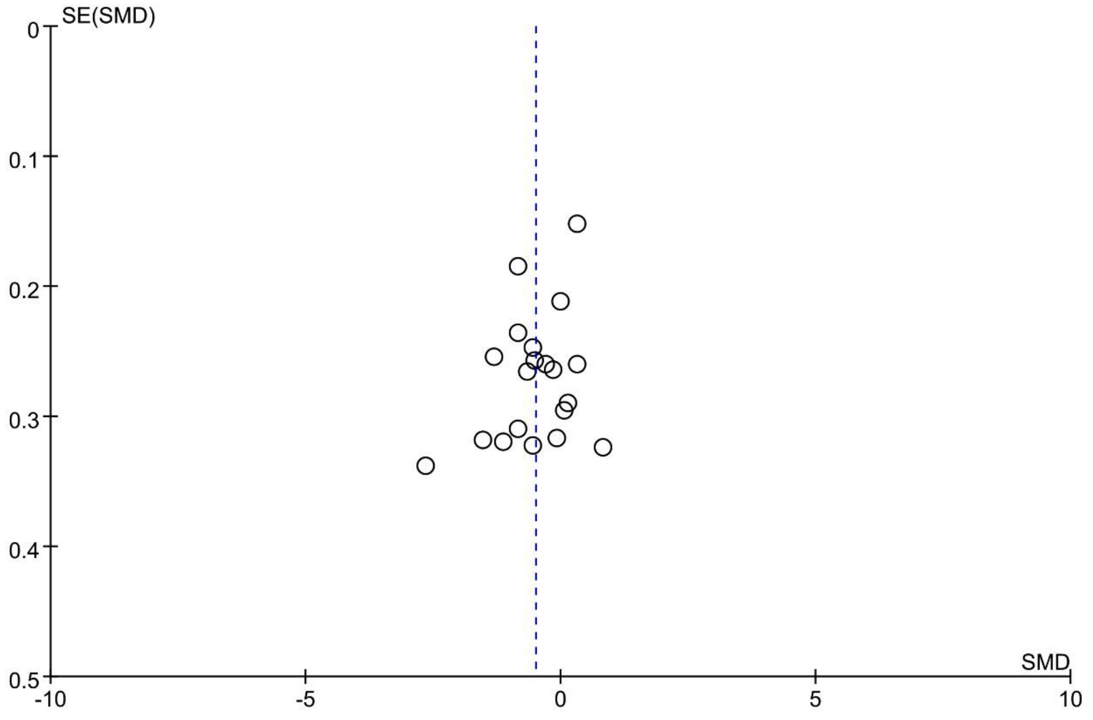

Supplement Figure 12. Funnel plot for LH level.

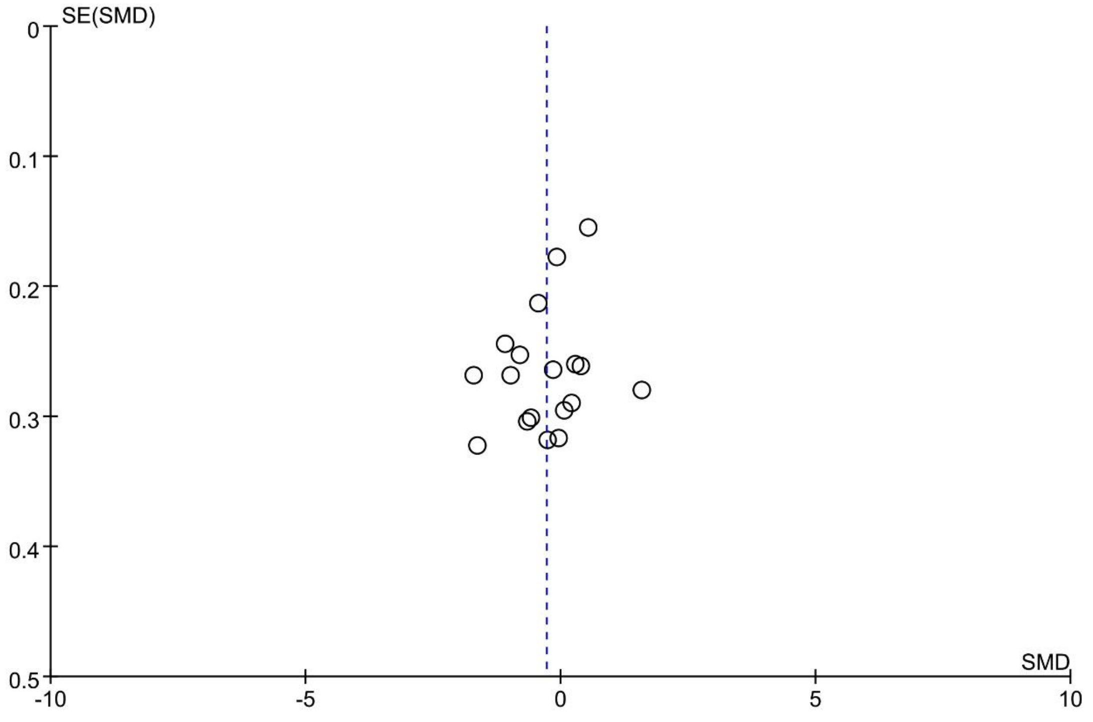

Supplement Figure 13: Sensitivity analysis for the total effective rate.

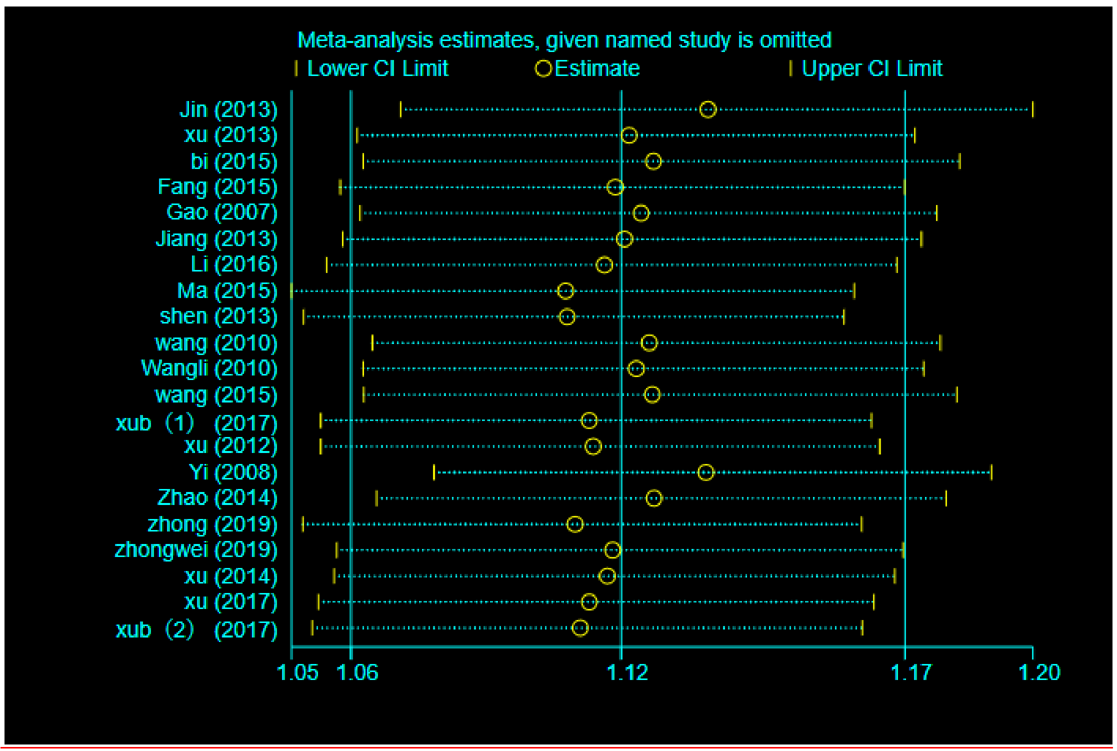

Supplement: Supplementary Materials — Supplement Digital: PubMed retrieval strategy. Supplement Table 1: composition of prescription in the included studies. Supplement Table 2: PRISMA checklist. Supplement Figure 1: subgroup analysis for total effective rate with different dosage forms. Supplement Figure 2: subgroup analysis for total effective rate with different duration treatment. Supplement Figure 3: subgroup analysis for E2 with different dosage forms. Supplement Figure 4: subgroup analysis for FSH with different dosage forms. Supplement Figure 5: subgroup analysis for LH with different dosage forms. Supplement Figure 6: subgroup analysis for E2 with different duration treatment. Supplement Figure 7: subgroup analysis for FSH with different duration treatment. Supplement Figure 8: subgroup analysis for LH with different duration treatment. Supplement Figure 9: funnel plot for total effective rate. Supplement Figure 10: funnel plot for E2 level. Supplement Figure 11: funnel plot for FSH level. Supplement Figure 12: funnel plot for LH level. Supplement Figure 13: sensitivity analysis for the total effective rate. [file 1789304.f1.pdf]
